# Deer thymosin beta 10 functions as a novel factor for angiogenesis and chondrogenesis during antler growth and regeneration

**DOI:** 10.1186/s13287-018-0917-y

**Published:** 2018-06-19

**Authors:** Wei Zhang, Wenhui Chu, Qingxiu Liu, Dawn Coates, Yudong Shang, Chunyi Li

**Affiliations:** 1 Institute of Special Animal and Plant Sciences, Chinese Academy of Agricultural Sciences, Changchun, 130112 Jilin People’s Republic of China; 2 State Key Lab for Molecular Biology of Special Economic Animals, 4899 Juye Street, Changchun City, 130112 Jilin People’s Republic of China; 3 Sir John Walsh Research Institute, Faculty of Dentistry, University of Otago, PO Box 56, Dunedin, 9054 New Zealand

**Keywords:** Thymosin beta 10, Deer antler, Stem cell, Angiogenesis, Blood vessel, Cartilage

## Abstract

**Background:**

Deer antlers are the only known mammalian organ with vascularized cartilage that can completely regenerate. Antlers are of real significance as a model of mammalian stem cell-based regeneration with particular relevance to the fields of chondrogenesis, angiogenesis, and regenerative medicine. Recent research found that thymosin beta 10 (TMSB10) is highly expressed in the growth centers of growing antlers. The present study reports here the expression, functions, and molecular interactions of deer TMSB10.

**Methods:**

The TMSB10 expression level in both tissue and cells in the antler growth center was measured. The effects of both exogenous (synthetic protein) and endogenous deer TMSB10 (lentivirus-based overexpression) on antlerogenic periosteal cells (APCs; nonactivated antler stem cells with no basal expression of TMSB10) and human umbilical vein endothelial cells (HUVECs; endothelial cells with no basal expression of TMSB10) were evaluated to determine whether TMSB10 functions on chondrogenesis and angiogenesis. Differences in deer and human TMSB10 in angiogenesis and molecular structure were determined using animal models and molecular dynamics simulation, respectively. The molecular mechanisms underlying deer TMSB10 in promoting angiogenesis were also explored.

**Results:**

Deer TMSB10 was identified as a novel proangiogenic factor both in vitro and in vivo. Immunohistochemistry revealed that TMSB10 was widely expressed in the antler growth center in situ, with the highest expression in the reserve mesenchyme, precartilage, and transitional zones. Western blot analysis using deer cell lines further supports this result. Both exogenous and endogenous deer TMSB10 significantly decreased proliferation of APCs (*P* < 0.05), while increasing the proliferation of HUVECs (*P* < 0.05). Moreover, deer TMSB10 enhanced chondrogenesis in micromass cultures and nerve growth as assessed using a dorsal root ganglion model (*P* < 0.05). Deer TMSB10 was proangiogenic using models of chicken chorioallantoic membrane, tube formation, and aortic arch assay. At the molecular level, endogenous deer TMSB10 elevated the expression of vascular endothelial growth factor (VEGF), VEGF-B, VEGF-C, and VEGF-D, and VEGFR2 and VEGFR3 in HUVECs (*P* < 0.05).

**Conclusions:**

Deer TMSB10, in contrast to its human counterpart, was identified as a novel stimulating factor for angiogenesis, cartilage formation, and nerve growth, which is understandable given that deer antlers (as the arguably fastest mammalian growing tissue) may require this extra boost during a period of rapid growth and regeneration.

**Electronic supplementary material:**

The online version of this article (10.1186/s13287-018-0917-y) contains supplementary material, which is available to authorized users.

## Background

**Fig. 1:**
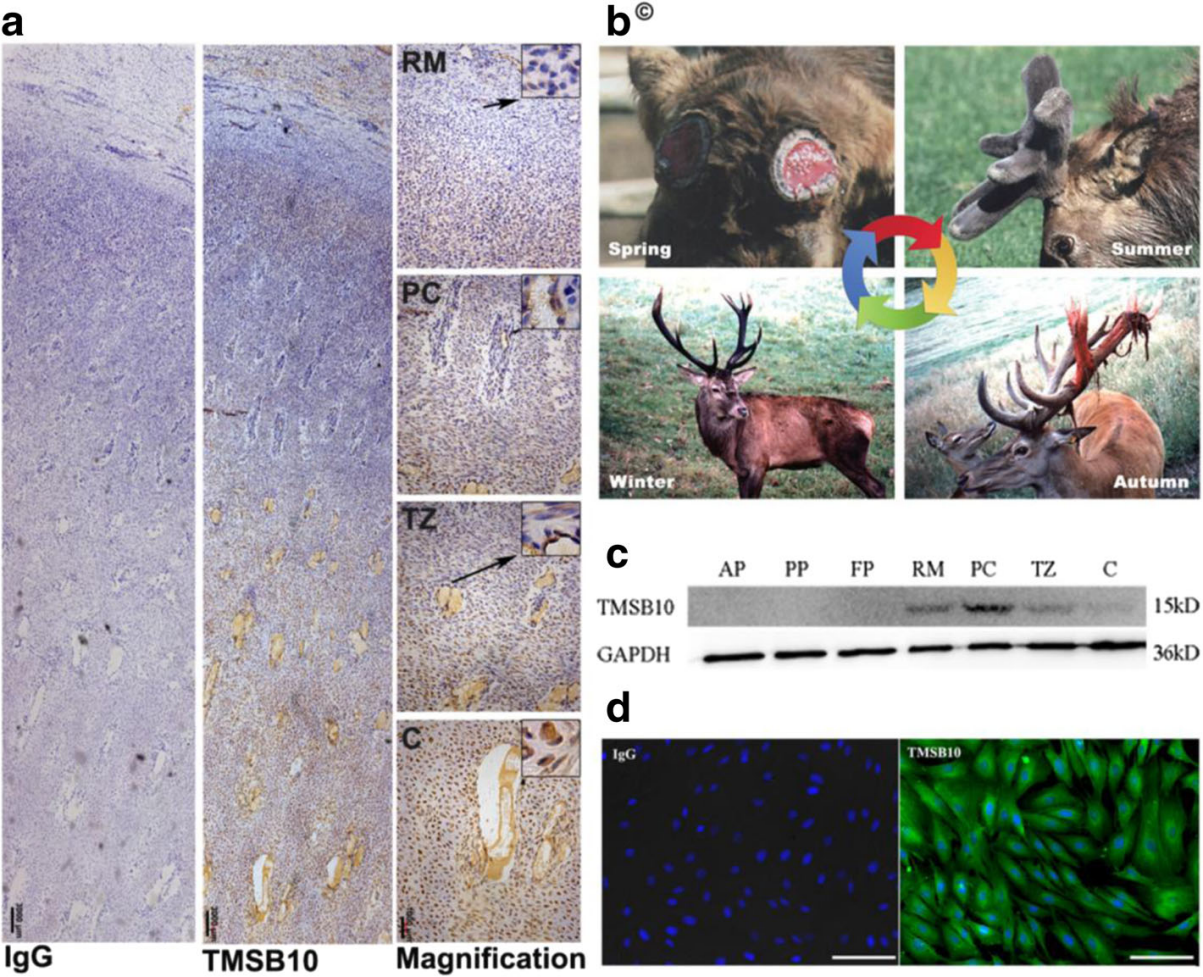
Immunohistochemical localization and expression of deer TMSB10 in the antler tip and in deer cell lines. **a** Deer TMSB10 protein in antler tip by immunohistochemistry. IgG shows rabbit IgG control, TMSB10 shows rabbit anti-TMSB10, Magnification shows enlarged view of TMSB10 section. Scale bar = 3000 μm for IgG and TMSB10; 1000 μm for magnification. **b** Antler regeneration cycle. In spring, hard antlers drop off from the pedicles, and antler regeneration immediately follows. Rapid antler growth occurs in summer. Growing antlers are enveloped with velvet skin. In autumn, antlers become fully calcified and velvet skin starts to shed. In winter, hard antlers are attached to their pedicles and subsequently cast in the next spring, which triggers a new round of antler regeneration. Copyright ©, reprinted from our previous works [1] with kind permission for educational use from *Frontiers in Bioscience*. **c** Protein expression levels of TMSB10 in deer cell lines as detected using Western blotting. **d** Representative images of TMSB10 protein in deer cell lines using immunofluorescence. Scale bars = 100 μm. AP antlerogenic periosteum, C cartilage, D dermis, FP facial periosteum, PC precartilage, PP pedicle periosteum, RM reserve mesenchyme, TZ transitional zone

Deer antlers are the only mammalian organs that can fully regenerate once lost from their pedicles (the permanent cranial bony protuberance from which an antler casts and regenerates), and hence offer the only opportunity to learn how mammalian epimorphic regeneration is regulated in nature (Fig.1b) [1]. The ultimate goal of regeneration research using model animals and/or organs such as antlers is to learn whether they can be used as a suitable model for regenerative medicine. Previous studies show that annual antler renewal is a stem cell-based epimorphic process driven by stem cells resident in the pedicle periosteum (PP). The PP is the direct derivative of antlerogenic periosteum (AP), a tissue from which a deer pedicle and an initial antler develop [2, 3]. Preliminary studies show that the process in early antler regeneration resembles that of wound healing over amputated mouse limb stumps [4]. Regeneration of antler cartilage, bone, skin, blood vessels, and nerves is achieved through both chemical induction and mechanical stimulation from the PP cell-derived progeny. Better understanding of the mechanisms of antler regeneration would undoubtedly contribute to the field of regenerative medicine, as well as provide potential application for treating some clinical diseases such as bone defects, osteonecrosis of the femoral head (ONFH), and bone fractures [5–8].

The beta-thymosin family members, which were originally identified from the thymus, have a number of subfamilies that include thymosin beta (TMSB)4, TMSB10, and TMSB15. They primarily function as actin sequestering proteins to inhibit actin polymerization and disrupt the formation of F-actin [9, 10]. Recent studies have focused on the role of the beta-thymosin family in the progression and metastasis of various cancers [11–13]. Furthermore, TMSB10 in humans, a 44-amino acid protein, exhibits diverse physiological functions beyond actin sequestration, including early organ development, cell apoptosis, proliferation, migration, angiogenesis, and tumorigenesis [14–17]. In a previous study, we found that TMSB10 was highly expressed in the growth center of the growing antler tip using a suppression subtractive hybridization cDNA library construction [18]. Subsequently, TMSB10 protein was localized in the different layers of the antler tip, thus supporting the previous results [19]. Thus far, the mechanisms underlying the functions of deer TMSB10 on angiogenesis and chondrogenesis during antler growth remain to be elucidated.

The aim of the present study was to investigate the functions of deer TMSB10 and hence to gain insights into the role of TMSB10 during antler growth as a unique mammalian model of epimorphic regeneration. To achieve this, we analyzed the differential expression of TMSB10 proteins in antler tip tissues and cell lines; we also used in-vitro and in-vivo models to evaluate the functions of TMSB10 for angiogenesis and chondrogenesis. Our results provide the first evidence that deer TMSB10 is proangiogenic, and its expression may contribute significantly to the blood vessel growth required during rapid antler regeneration. Understanding TMSB10 and other relevant molecules that regulate the formation of antlers may provide valuable insights into the development of future treatment options in the rapidly developing field of regenerative medicine.

## Methods

All experiments were performed in accordance with the guidelines and study protocols of the Animal Ethics Committee of the Institute of Special Animals and Plants, Chinese Academy of Agricultural Science (ethics number: CAAS201415 for deer; CAAS201519 for rats; and CAAS201611 for chicken embryos). All efforts were made to minimize the number of animals used.

### Reagents and antibody

Recombinant vascular endothelial growth factor (VEGF; 100–20), recombinant nerve growth factor (β-NGF; 450–01), recombinant human transforming growth factor (TGFβ1; 100–21), and recombinant human hepatocyte growth factor (HGF; 100-39H) were purchased from Peprotech Inc. (USA); basic fibroblast growth factor (bFGF; 62031–54-3) was purchased from Dalian Meilunbio (China); dexamethasone (D1756), l-ascorbic acid 2-phosphate (A4403-100MG), and l-proline (P0380-10MG) was purchased from Sigma (USA); and ITS Premix Universal Culture Supplement was purchased from Corning (USA, 354351). Antibodies used in this study are listed in Table 1.

**Table 1:** Antibodies

Terms	Source	Type	Company	Catalog number	Dilution
Thymosin beta 10 (TMSB10)	Rabbit	Polyclonal antibody	Abcam, USA	Ab14338	WB: 1:2000; IHC: 1:1000; IF: 1:500 (whole antiserum)
Vascular endothelial growth factor-A (VEGFA)	Rabbit	Polyclonal antibody	Boster, China	BA0407	WB: 0.4 μg/ml (1:500)
Vascular endothelial growth factor-B (VEGFB)	Rabbit	Polyclonal antibody	Elabscience, China	E-AB-33212	WB: 1 μg/ml (1:1000)
Vascular endothelial growth factor-C (VEGFC)	Rabbit	Polyclonal antibody	Boster, China	BA0548	WB: 0.4 μg/ml (1:500)
Vascular endothelial growth factor-D (VEGFD)	Rabbit	Polyclonal antibody	Elabscience, China	E-AB-33213	WB: 1 μg/ml (1:1000)
Vascular endothelial growth receptor 1 (FLT1)	Rabbit	Polyclonal antibody	Proteintech, China	13687–1-AP	WB: 0.26 μg/ml (1:1000)
Vascular endothelial growth factor receptor 2 (KDR)	Rabbit	Polyclonal antibody	Bioss, China	Bs-10412R	WB: 2.5 μg/ml (1:400)
Vascular endothelial growth factor receptor 3 (FLT4)	Rabbit	Polyclonal antibody	Proteintech, China	20712–1-AP	WB: 0.83 μg/ml (1:500)
Rabbit IgG-Isotype control	Rabbit		Abcam, USA	Ab172730	IHC: 1.78 μg/ml (1:1000) IF: 3.56 μg/ml (1:500)
Glyceraldehyde 3 phosphate dehydrogenase (GAPDH)	Mouse	Monoclonal antibody	Proteintech, China	60004–1-Ig	WB: 0.5 μg/ml (1:2000)
HRP-conjugated goat anti-rabbit IgG (H + L)			Beyotime, China	A0208	WB: 1:2000 IHC: 1:500
HRP-conjugated goat anti-mouse IgG (H + L)			Beyotime, China	A0216	WB: 1:2000
Alexa Fluor 488 labeled goat anti-rabbit IgG (H + L)			GeneCopoeia, USA	L110B	IF: 1:250

*HRP* horseradish peroxidase, *IF* immunofluorescence, *IHC* immunohistochemistry, *WB* Western blotting

### Antler growth center tissue sampling and immunohistochemistry

Three-year-old sika deer (*Cervus Nippon*) (*n* = 3) were used in this study and one antler was collected from each animal (ethics number: CAAS201415). The antler was harvested using traditional Chinese antler harvesting procedures at around 35 days of growth, which is approximately half way through the antler growth stage. The distal 5-cm tip of the main beam of each antler was collected and sectioned sagittally along the median plane [20]. Samples were then fixed in 4% paraformaldehyde (Sigma, China, 158,127-500G) and processed for histology as previously described [21]. Immunohistochemistry was performed on the sagittal sections of paraffin-embedded antler tips. After being deparaffinized with xylene, endogenous peroxidase activity was quenched with 0.3% H_2_O_2_, and antigen retrieval was performed with 0.01 M citric acid at 95 °C for 20 min. Sections were then incubated with anti-TMSB10 antibody or anti-rabbit IgG (isotype control) overnight at 4 °C (antibody information is shown in Table 1). After warming and rinsing, sections were incubated with the appropriate horseradish peroxidase (HRP)-conjugated secondary antibody for 30 min at 37 °C. Finally, sections were stained with diaminobenzidine (Maixin, China, DAB-0031) and counterstained with hematoxylin.

### Immunofluorescence and morphological staining

Deer cells (reserve mesenchyme (RM)) were fixed with 4% paraformaldehyde and blocked by incubation in 3% bovine serum albumin (BSA) and 0.1% Triton X-100 (Sigma, USA, T8787-100ML) in phosphate-buffered saline (PBS) for 1 h at room temperature, followed by incubation with the primary antibody overnight at 4 °C. The next day, the cells were incubated with secondary antibody for 30 min followed by DAPI (blue) staining of the nuclei. The primary and secondary antibodies used in this process are listed in Table 1. Rabbit IgG replaced the primary antibody for the isotype-matched controls. Morphology (microtubule or microfilament) of the AP and human umbilical vein endothelial cells (HUVECs) was conducted using TRITC-Phalloidin (ThermoFisher, USA, R415) and Actin-Tracker Green (Beyotime, China, C1033), according to the manufacturers’ instructions. All the images were captured using an inverted microscope with a digital CCD camera (EVOS, ThermoFisher, USA).

### Cell culture

The deer cell lines used in this study were: antlerogenic periosteum (AP), pedicle periosteum (PP), facial periosteum (FP), reserve mesenchyme (RM), precartilage (PC), transitional zone (TZ), and cartilage (C). All cell lines were harvested from three male sika deer (at 8 months for AP, 2 years for PP and FP, and 3 years for RM, PC, TZ, and C) (Additional file 1: Figure S1) [22] (ethics number: CAAS201415). HUVECs were purchased from ScienCell (USA, 8000) and maintained at 37 °C under 5% CO_2_ in RPMI-1640 medium (GIBCO, USA, 21875091) supplemented with 10% fetal bovine serum (FBS; GIBCO, USA, 10099141), 100 U/ml penicillin, and 100 μg/ml streptomycin (Corning, USA, 30–002). Primary cultures of deer cells were cultured under similar conditions; however, the base medium was Dulbecco’s modified Eagle’s medium (DMEM; GIBCO, USA, 11965092) [23]. Each cell type was passaged using 0.08% trypsin (Life technologies, USA, 27250018) and stored in liquid nitrogen in the freezing medium (FBS + 10% dimethyl sulfoxide (DMSO)). The cells used in this study for all experiments were between 4 and 10 passages.

### Amplification of full-length deer TMSB10

Total RNA was extracted from cultured RM cells using a PureLink™ RNA mini kit (Life Technologies, USA, 12183018A) according to the manufacturer’s instructions. Reverse transcription (RT) was performed using 1 μg RNA and Primescript RT Reagent Kit (TaKaRa, Dalian, China, R023A). Gene specific primers (Additional file 2: Table S1; TMSB10-RT) were used to amplify the coding region of the full-length cDNA of TMSB10 using an Eppendorf Mastercycler (Germany). Amplification was carried out under the following conditions: 5-min activation step at 95 °C; 35 cycles of 10 s at 95 °C, 5 s at 55.5 °C, and 1 min at 72 °C. The polymerase chain reaction (PCR) products were gel-purified using a QIAEX II gel extraction system (QIAGEN, Germany, 20,021) and cloned into the pMD-19 T vector (TaKaRa, Dalian, China, 6013). At least three independent clones of each amplicon were selected and sequenced by simultaneous bidirectional DNA sequencing (Sangon Biotech Shanghai Co. Ltd.). The coding amino acid sequence was predicted and described using DNAman software (Version 8.0.8.789) and NCBI (www.ncbi.nlm.nih.gov). The structure of deer TMSB10 protein was analyzed using homologous modeling technologies (SWISS-MODEL, www.swissmodel.expasy.org).

### Vectors and preparation of recombination lentivirus

The deer TMSB10 coding region was amplified using the following gene-specific primers where the recombination site of the vector has been shaded (Additional file 2: Table S1; TMSB10-Clone). The gel-eluted amplification products (162 bp) were cloned into the pLVX-ires-mCherry-puro plasmid using a ClonExpress II One Step Cloning Kit (Vazyme Biotech Co. Ltd., Nanjing, China, C112–01) according to the manufacturer’s instructions. Plasmid DNA was then isolated with the Concert Rapid Plasmid Miniprep System (Omega, USA, D6943–01) and the sequence verified by simultaneous bidirectional DNA sequencing. Production of lentivirus was carried out according to previous reports [24, 25]. Briefly, the lentiviral vector system, consisting of pLVX-ires-mCherry-puro/-TMSB10, pSPAX2, and pMD2.G plasmids, was cotransfected into 293 T cells using X-tremeGENE DNA transfection reagent (Roche, Germany, 06366236001) according to the manufacturer’s protocol. Virus-containing supernatants were collected at 24 h and 48 h after transfection, pooled together, concentrated by centrifugation at 1500 g using the Lenti-X concentrator (Clonetech, Japan, 631232), and stored at −80 °C.

### Lentiviral infection of antlerogenic periosteal cells (APCs) and HUVECs

The APCs and HUVECs were seeded in six-well culture plates (Corning Coster, NY, USA) and, after reaching 50% confluence, the cells were infected with lentivirus as described by Guo et al. [26]. Briefly, the medium was replaced with lentiviral-vector supernatants (TMSB10 or empty carrier) in the presence of 10 μg/ml polybrene (Millipore, USA, TR-1003-50UL). Forty-eight hours after infection, expression of the antibiotic resistance gene was initiated. The cells were treated and enriched using puromycin (Sigma, Shanghai, China, p8833) at an optimized concentration of 2.5 μg/ml for APCs and 3 μg/ml for HUVECs for a period of 2 weeks (Additional file 3: Figure S2A, B). APCs or HUVECs overexpressing deer TMSB10 (ODT) were named as APCs-ODT or HUVECs-ODT.

### Preparation of exogenous thymosin beta 10 protein

Deer and human exogenous TMSB10 protein (eTMSB10) was synthesized via solid-phase peptide synthesis [27]. The proteins were then purified through high-performance liquid chromatography (HPLC) and the amino acid sequence was confirmed by electrospray ionization mass spectrometry (ESI-MS) following the manufacturer’s instructions (Sangon Biotech Co. Ltd., China). Purity for both deer and human eTMSB10 was > 98.00% (98.25% for deer and 98.13% for human) (Additional file 4: Figure S3). The peptides were then redissolved in PBS (pH 7.2) with 0.1% BSA (Sigma, China, B2064-10G) and stored at −80 °C.

### In vitro cytotoxicity MTT assay

The relative proliferation rates of the APCs and HUVECs were measured using an MTT assay as previously described [28]. In brief, cells were collected from the logarithmic growth phase and seeded in triplicate into 96-well plates at a density of 2500 and 5000 cells/well for APCs and HUVECs, respectively. The cells were cultured in medium supplemented with deer eTMSB10 protein at 50, 100, and 150 ng/ml for 24 h, 36 h, and 72 h (medium was renewed daily). In addition, a growth curve of APCs-ODT and HUVECs-ODT and control vectors were measured. At each time point, cells were incubated in medium containing 20 μl MTT/well for 4 h (Sigma-Aldrich, USA, M2128-100MG). DMSO (150 μl; Amresco, USA, 0231) was added to solubilize the formazan crystals and the optical density at 570 nm (OD_570_) was measured using a spectrophotometer (Tecan, Switzerland). Data are presented as a percentage of the control wells. Mean ± standard deviation (SD) is shown for *n* = 3 independent experiments (*P < 0.05, **P < 0.01).

### In vitro cell migration assay

HUVEC migration was investigated using a modified Transwell assay (Costar, NY, USA; pore size 8 μm, 3422) in 24-well plates [29]. Deer eTMSB10 at 50, 100, and 150 ng/ml in 700 μl culture medium containing 5% FBS was placed in the bottom reservoir of each chamber. Cultured HUVECs were harvested using trypsin after serum starvation for 12 h, washed twice in RPMI-1640 (without FBS), and resuspended in serum-free medium. Cells were plated at a density of 1 × 10^4^ cells/200 μl in the upper wells of each chamber and incubated for 12 h at 37 °C under a humidified atmosphere of 5% CO_2_/95% air. HUVECs-ODT were treated in the same way without the addition of deer eTMSB10. The cells were fixed in 100% methanol for 20 min and stained in 0.1% crystal violet in PBS (v/v) for 15 min at room temperature. Cells on the upper side of the filters were removed with cotton tipped swabs, and the filters washed three times with PBS. Cells that migrated through the filters were counted under a microscope, the crystal violet staining was extracted using 33.3% acetic acid (v/v), and the OD_570_ measured using a spectrophotometer (Tecan, Switzerland). Experiments in this study were repeated three times.

### In vitro wound healing assay

The wound healing assay was carried out using wound healing culture inserts (IBIDI technologies, 80,206). Each reservoir (0.22 cm^2^) was loaded with 70 μl medium containing 3 × 10^5^ cells/ml for APCs or 5 × 10^5^ cells/ml for HUVECs, and incubated in medium supplemented with 10% FBS for 24 h at 37 °C under 5% CO_2_/95% air. Once the cells reached confluence (24 h) the inserts were removed and the wells were filled with 400 μl/well of medium (1% FBS for AP cells, and serum-free for HUVECs) supplemented with deer eTMSB10 (100 ng/ml) or VEGF (20 ng/ml) as a positive control. The time taken by the cells to fill the gap was observed under an inverted microscope and measurements taken after 24 h. Results are reported as the mean ± SD, *n* = 3 independent experiments (*P < 0.05, **P < 0.01).

### In vitro tube formation assay

The effects of deer TMSB10 on angiogenesis were measured using an endothelial cell tube formation assay with a Matrigel matrix as previously described [30]. In brief, growth factor-reduced Matrigel (50 μl at 10 mg/ml; BD technologies, 354,230) was added into a 96-well plate and polymerized for 45 min at 37 °C. Unmodified HUVECs (1.5 × 10^4^ cells) or HUVECs-ODT were seeded on the surface of the Matrigel and cultured in serum-free medium (RPMI-1640) supplemented with or without eTMSB10 protein (50, 100, and 150 ng/ml) or with VEGF (20 ng/ml) as a positive control. Cells were then incubated for 6 h at 37 °C with 5% CO_2_. Morphologic changes were photographed at 40× magnification and tube formation determined using an inverted microscope with a digital CCD camera (EVOS, ThermoFisher, USA).

### In vitro rat aortic ring assay

Rat aortic ring assays were performed as previously published [30]. In brief, Wistar rats (8–12 weeks old) were deeply anesthetized and sacrificed according to local ethical guidelines (ethics number: CAAS201519). Aortic vessels were collected and detached from the spine using sterile microdissection forceps starting from the diaphragm toward the heart. The aortic vessels were then washed three times with ice-cold RPMI-1640 medium containing antibiotics and cut into equal sections ~ 1 mm long with the aid of a slice cutting machine as invented by our research group [31]. Aortic arch slices were incubated with different concentrations of deer eTMSB10 protein (50, 100, and 150 ng/ml). Unconditioned medium or medium with VEGF (20 ng/ml) as the control were also added onto the polymerized Matrigel for 5 days, after which the medium was refreshed every 2 days. Morphologic changes were photographed for quantification. Data represent mean ± SD of three experiments (*P < 0.05, **P < 0.01).

### Chicken embryo chorioallantoic membrane (CAM) assay

CAM assays were performed as described previously [32] with slight modifications. Fertilized White Leghorn chick eggs were incubated at 38 °C under constant high humidity (85%) (ethics number: CAAS201611). On the third day of incubation, a square (1 cm^2^) window was made on the shell after removal of 2–3 ml albumen to detach the developing CAM from the shell. The window was then sealed with a wrap of the same size, and the eggs were returned to the incubator. On day 6, sterilized filter-paper (6 mm in diameter; Millipore, USA, 14250) that had been dried after soaking in the test materials of deer eTMSB10 (1, 5, and 10 μg), human eTMSB10 (1, 5, and 10 μg), HGF (1 μg), and bFGF (4 μg) all dissolved in 20 μl 0.1% BSA in PBS (pH 7.2) were positioned on each CAM under sterile conditions. On day 10, the CAMs were fixed with 4% paraformaldehyde in PBS (pH 7.2–7.4) in situ and kept at 37 °C for 2 h. Afterwards, the discs were removed and a large area around them dissected away, placed on a glass slide, and the number of blood vessels present were photographed and counted (Leica, Germany). The number of newly formed blood vessels was quantified using the method of Zhou et al. [33]. Briefly, newly formed blood vessels (1 mm around the carrier) were identified by the angle of vessel in relation to the carrier center, and newly formed vessels less than or equal to 45 degrees were counted, while vessels less than 0.05 mm in diameter were excluded from counting. The counting of vessels was performed using a double-blinded methodology. The number of vessels was expressed as a percentage increase or decrease from the control disc (mean ± SD).

To test the effects of AP cells with deer TMSB10 overexpression on angiogenesis in the CAM assay, the carriers (filter-papers) were replaced by Matrigel diluted with serum-free DMEM (Matrigel:DMEM = 1:2.5) with AP cells (1 × 10^6^ cells/100 μl) in a total volume of 100 μl on ice and polymerized for 60 min at 37 °C.

### In vitro chondrocyte induction of APCs using micromass culture

Chondrogenic differentiation experiments were performed using micromass cultures following the method reported elsewhere [34]. Briefly, the APCs-ODT were cultured in T75 flasks, trypsinized when reaching 85–90% confluence, and resuspended in chondrogenic medium (high-glucose DMEM, 10 ng/ml recombinant human TGFβ1, 0.1 μM dexamethasone, 50 μg/ml l-ascorbic acid 2-phosphate, 40 μg/ml l-proline, and 1% ITS) at a concentration at 1 × 10^8^ cells/ml. Aliquots of 100 μl of cell suspension were seeded in the center of each well of a six-well plate (Nunc, Denmark) and incubated for 3 h at 37 °C to facilitate adherence of the cells. Thereafter, 2 ml chondrogenic medium was gently added into each well around the forming cell aggregate. The induction medium was subsequently replaced every 2 days and observed daily under phase-contrast microscopy. The resultant cell nodules were harvested 3 weeks after initial seeding. Micromass proteoglycan content was visualized using Alcian Blue (pH 2.5) on 6-μm sections through the nodules.

### Dorsal root ganglia (DRG) outgrowth assay

The procedure for culturing DRG was based on previously published research [35]. Briefly, DRGs were extracted from fetal rats (Wistar, E16) using microdissection forceps under a dissecting microscope (Leica, Germany) and immediately transferred into 35-mm petri dishes filled with ice-cold PBS and antibiotics (ethics number: CAAS201519). DRGs were cultured in serum-free neurobasal medium (Invitrogen, USA, 21103049) containing antibiotics and B-27™ serum-free supplement (Invitrogen, USA, 17504044) in 96-well plates coated with poly-l-lysine (10 μg/mL; Sigma, USA, P4707) and cultured at 37 °C with 5% CO_2_. Deer eTMSB10 protein (50, 100, 150 ng/ml) or NGF (50 ng/ml) were added into the cultures for 5 days (medium refreshed every 2 days). Morphologic changes in the DRGs were photographed with a digital CCD camera, at 40× magnification after 2 and 5 days.

### Western blotting

Total protein was extracted from the deer cells lines and HUVECs-ODT cells with lysis buffer (Beyotime, China, P0013B) containing protease inhibitors (Sigma, USA, P8340-1ML) and phosphatase inhibitors (KyGene, China, KGP250), and protein concentrations were determined via Bradford [36]. Proteins (60 μg) were resolved using 12% polyacrylamide-SDS gels (15% for TMSB10) and transferred to polyvinylidene difluoride (PVDF) membranes (Millipore, MA, USA, ISEQ00010; 0.22 μm for TMSB10) using standard methods. Membranes were then blocked with 5% (w/v) nonfat dry milk (NFDM; BD biosciences, USA, 232100) in TBS-T (Solarbio, China, T1080) on a rotating shaker for 2 h at room temperature. The membranes were probed with specific primary antibodies (Table 1) overnight at 4 °C, washed three times (15 min each) using TBS-T, and incubated with HRP-conjugated secondary antibodies at room temperature for an additional 1.5 h. The levels of proteins were visualized using an ECL system (ThermoFisher, USA, 34077; SAGECREATION, minichemi 610 Plus, China). Western blots were performed in triplicate and the target protein bands were quantified by scanning densitometry using Image-pro plus (version 6.0) processing software [37] and normalized to the signal intensity of GAPDH.

### Homology modeling and molecular dynamics simulation of TMSB10 structure

Deer TMSB10 protein was sequenced and human TMSB10 protein sequence obtained from NCBI (www.ncbi.nih.gov, GeneBank: AIC55540.1). Homology modeling of the three-dimensional (3D) structures was constructed using the bovine TMSB10 template (PDB ID: 1HJ0) and homology modeling software (SWISS-MODEL, www.swissmodel.expasy.org).

Molecular dynamics (MD) simulations were performed on an HP DL980 server using the AMBER14 software package [38]. The ff14sb force field [39] was used for energy minimization and MD simulations since it has been widely used for the conformational analysis of proteins. The charges of deer and human TMSB10 models were neutralized by the tleap module of AMBER14. The 12 Å explicit solvent model of TIP3P water box [40] was used between the TMSB10 surface and the edge of the water box. Energy minimization (EM) was performed before the MD simulations. With 500 kcal/mol^1^/Å^2^ restraint on TMSB10, a minimization of 1000-step steepest descent (SD) and 1000-step conjugate gradient (CG) were carried out. Subsequently, without any restraint, 3000-step SD and 4000-step CG minimization was carried out. A 500 ps heating simulation was performed from 0 to 300 K. A weak constraint force constant value (10 kcal/mol^1^/Å^2^) was used for TMSB10. After that, without any constraint, an NPT ensemble under 1 atm and 300 K was adopted for a 100-ns equilibrium simulation. Periodic boundary conditions were used on the whole system. The nonbonded cutoff value interaction was 12 Å. The long-range electrostatic interactions were calculated using the Particle Mesh Ewald (PME) method [41]. The bond lengths of hydrogen-heavy atoms were constrained using the SHAKE algorithm [42]. The time step was 2.0 fs. A simple Leapfrog integrator was used with the collision frequency of 1 ps^−1^. A Langevin thermostat was adopted and the relaxation time was 2.0 ps for the barostat bath. After the MD simulation, the refined structure was inspected using Procheck [43] and Profile-3D [44]. Each system was simulated for 100 ns and trajectories were sampled at a 10-ps interval. The structural analysis was performed using the root-mean-square deviation (RMSD) and Cα-root-mean-square fluctuation (RMSF).

### Statistical analysis

All experiments in this study were performed at least in triplicate for each control and treatment group (two repeats in CAMs assay for APCs). The numeric data are expressed as the mean ± SD. Differences between groups were evaluated using Student’s *t* test. *P* < 0.05 was considered statistically significant. GraphPad Prism 5 (version 5.01) (www.graphpad.com) was used to display and analyze the data.

## Results

### Characterization of deer TMSB10

The coding region of deer TMSB10 was cloned and sequenced (Additional file 5: Figure S4A). It consisted of 129 bps encoding a 42-amino acid protein. Deer TMSB10 has a predicted molecular weight of 4.82 kDa and a theoretical isoelectric point of 6.65. Its three-dimensional structure (Additional file 5: Figure S4B) had two helices. SignalP analysis (www.cbs.dtu.dk/services/SignalP/) showed that this protein did not have a probable signal peptide. The deduced amino acid sequence showed that deer TMSB10 had high sequence similarity to TMSB10 proteins from other species: 93%, 93%, 99%, and 100% to the human, mouse, bovine, and sheep, respectively (Additional file 5: Figure S4C).

### Tissue/cell-specific expression of deer TMSB10 proteins

Deer TMSB10 protein in the antler growth center was localized using immunohistochemistry, and from distal to proximal (dermis (D), RM, PC, TZ, to C) showed a characteristic pattern; i.e., low-high-low (Fig. 1a). The expression level of deer TMSB10 protein in the deer cell lines (different layers within the antler growth center and AP) were investigated using Western blot analysis (Fig. 1c). A localized band of an unexpected size (15 kDa) was present in the deer cell lines corresponding to a TMSB10 protein (Fig. 1c). Immunofluorescent results showed that deer TMSB10 protein was mainly localized in the cytoplasm of deer cells (Fig. 1d).

### Effects of both deer and human eTMSB10 on angiogenesis in vivo

**Fig. 2:**
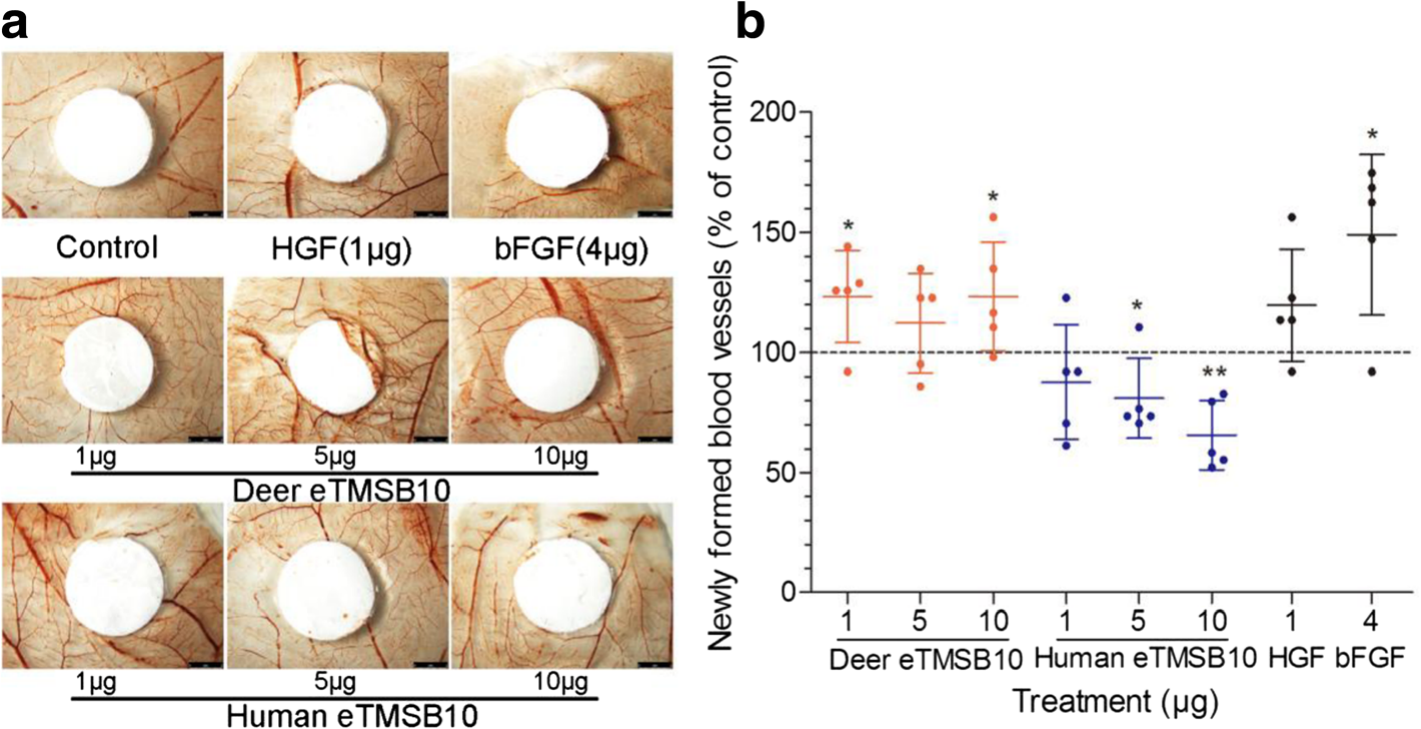
CAM assays investigating both deer and human eTMSB10 at 1, 5, and 10 μg and controls of hepatocyte growth factor (HGF; 1 μg) and basic fibroblast growth factor (bFGF; 4 μg). **a** Representative pictures of CAMs tested with eTMSB10 and control agents. **b** Quantitation of number of newly formed blood vessels in the CAMs. Newly formed blood vessels in five eggs per group were counted using a blinded methodology. Control were set at 100%. Human eTMSB10 protein significantly inhibited, while deer eTMSB10 protein significantly promoted, the formation of newly formed blood vessels. Scale bar = 2 mm

TMSB10 protein was highly expressed in the PC layer (the zone where blood vessel formation occurs during antler growth [45]), and also the blood vessel walls in the other layers of the growth center. It was thus speculated that deer TMSB10 might play an important role in angiogenesis during antler growth. A previous study reported that human synthesized TMSB10 (eTMSB10) exhibited an inhibitory effect on angiogenesis when tested using CAM assays [46]. CAM assays were conducted here to confirm the previous findings and the results showed that human eTMSB10 reduced the numbers of newly formed blood vessels, while deer eTMSB10 significantly promoted the formation of newly blood vessels (Fig. 2) (*P* < 0.05). To our knowledge, this is the first evidence showing that deer eTMSB10 promotes angiogenesis in vivo. HGF at 1 μg and bFGF at 4 μg were used as positive controls and significantly increased the number of blood vessels (*P* < 0.05 for bFGF).

### Effects of deer TMSB10 on cell proliferation, cytoskeletal formation, and chondrogenesis of the APCs

**Fig. 3:**
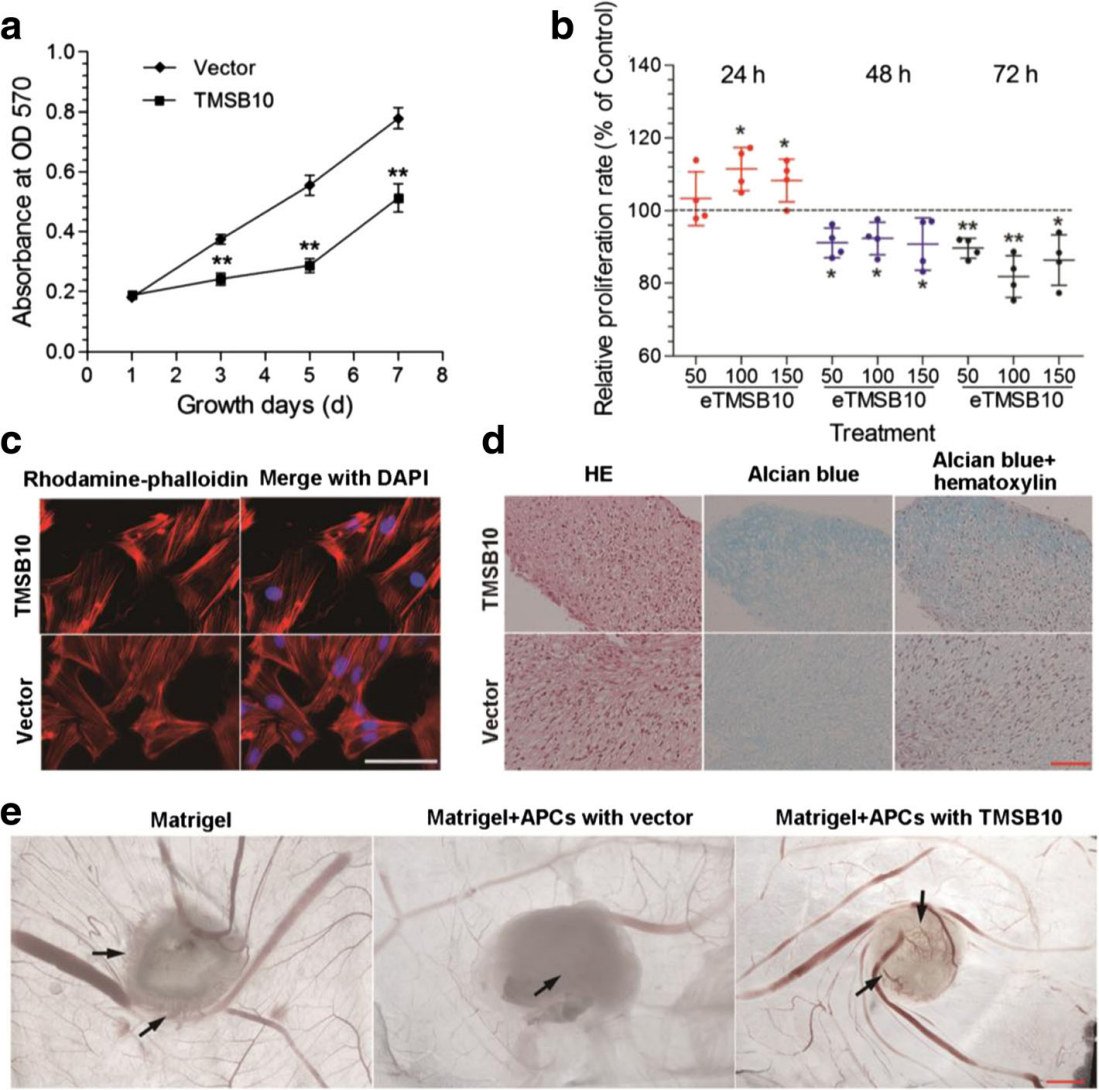
Effects of deer TMSB10 on cell proliferation, cytoskeletal formation, chondrogenesis, and angiogenesis of antlerogenic periosteum cells (APCs). **a** Determination of proliferation over 7 days of APCs overexpressing deer TMSB10, measured with an MTT assay. **b** Proliferation of APCs exposed for 24, 48, and 72 h to exogenous deer TMSB10 (eTMSB10: 50, 100, or 150 ng/ml) compared with control wells with no deer eTMSB10. Cell proliferation (MTT assay) is presented as the percentage of viability of control cells (control set at 100%). Results are reported as the mean ± SD, *n* = 4 independent experiments. *P < 0.05, **P < 0.01, versus control. **c** Determination of F-actin morphology of APCs overexpressing deer TMSB10 using the TRITC-Phalloidin. Overexpression of the gene slightly reduced the numbers of F-actin-positive fibers in the APCs. Scale bar = 200 μm. **d** Chondrogenic differentiation of APCs overexpressing deer TMSB10 grown as micromass cultures. The cells were treated with differentiation media for 3 weeks. The Alcian Blue staining depth of the resultant nodules was increased in the cells with deer TMSB10 overexpression compared with the control group. Scale bar = 100 μm. **e** CAM assay for APCs overexpressing deer TMSB10. APCs overexpressing deer TMSB10 and with empty vector were loaded on the CAMs with Matrigel for 4 days. Results showed that new blood vessels were most noticeably induced by APCs overexpressing deer TMSB10, where vessels were evident both at the border and inside the Matrigel (arrows). Scale bar = 2 mm. HE hematoxylin an eosin, OD optical density

To determine the biological roles of deer TMSB10, we selected two cell lines, both of which have no TMSB10 expression: one was APCs (antler stem cells, from which pedicles and antlers are differentiated) (Fig. 1c; Additional file 6: Figure S5), and the other was HUVECs. TMSB10 expression was induced and the angiogenic and chondrogenic activities of these cells examined. We firstly constructed APCs that stably expressed deer TMSB10. Western blot analysis was performed to measure the protein levels of deer TMSB10 expression (Additional file 3: Figure S2; Additional file 6: Figure S5). MTT proliferation assays revealed that overexpression of deer TMSB10 significantly decreased the proliferation rate of the APCs compared with the vector-only control over a 3–7 day period (Fig. 3a) (*P* < 0.05). In addition, the proliferation of normal APCs was examined after deer eTMSB10 was added to the medium. eTMSB10 significantly increased APC proliferation at 24 h compared with controls for the 100 and 150 ng/ml doses (*P* < 0.05), but had no effect at the 50 ng/ml dose (*P* > 0.05); however, the number of APCs was reduced at the 48 and 72 h time points for all doses (Fig. 3b) (*P* < 0.05). To investigate the effects of deer TMSB10 on the activity of F-actin assembly in the APCs, a TRITC phalloidin assay was performed and found that overexpression of TMSB10 slightly reduced the numbers of F-actin-positive fibers in the APCs (Fig. 3c). In addition, 100 ng/ml of deer eTMSB10 had no significant effects on the migration of the APCs using IBIDI cell migration technology (Additional file 7: Figure S6) (*P* > 0.05). We further evaluated the effects of deer TMSB10 on chondrogenesis using the APCs in micromass culture. As shown in Fig. 3d, the Alcian Blue staining intensity of the resultant nodules was increased in APCs-ODT compared with the control group. To further confirm the role of deer TMSB10 in angiogenesis during the period of rapid antler growth, we investigated the effects of the protein when overexpressed in AP cells using an in-vivo CAM assay. Results showed that Matrigel alone induced formation of some new blood vessels which were small and along the edge of the carrier (Matrigel) (Fig. 3e; arrows). New blood vessels were most noticeably induced by APCs-ODT (Fig. 3e; arrows), where vessels were evident both at the border and inside the Matrigel. In addition, the level of tumor necrosis factor (TNF)α was elevated in the culture medium of APCs-ODT compared with the vector alone (Additional file 8: Figure S7) (*P* < 0.05).

### Effects of deer TMSB10 on cell proliferation, tube formation, and cell motility of HUVECs, as well as on DRG outgrowth

**Fig. 4:**
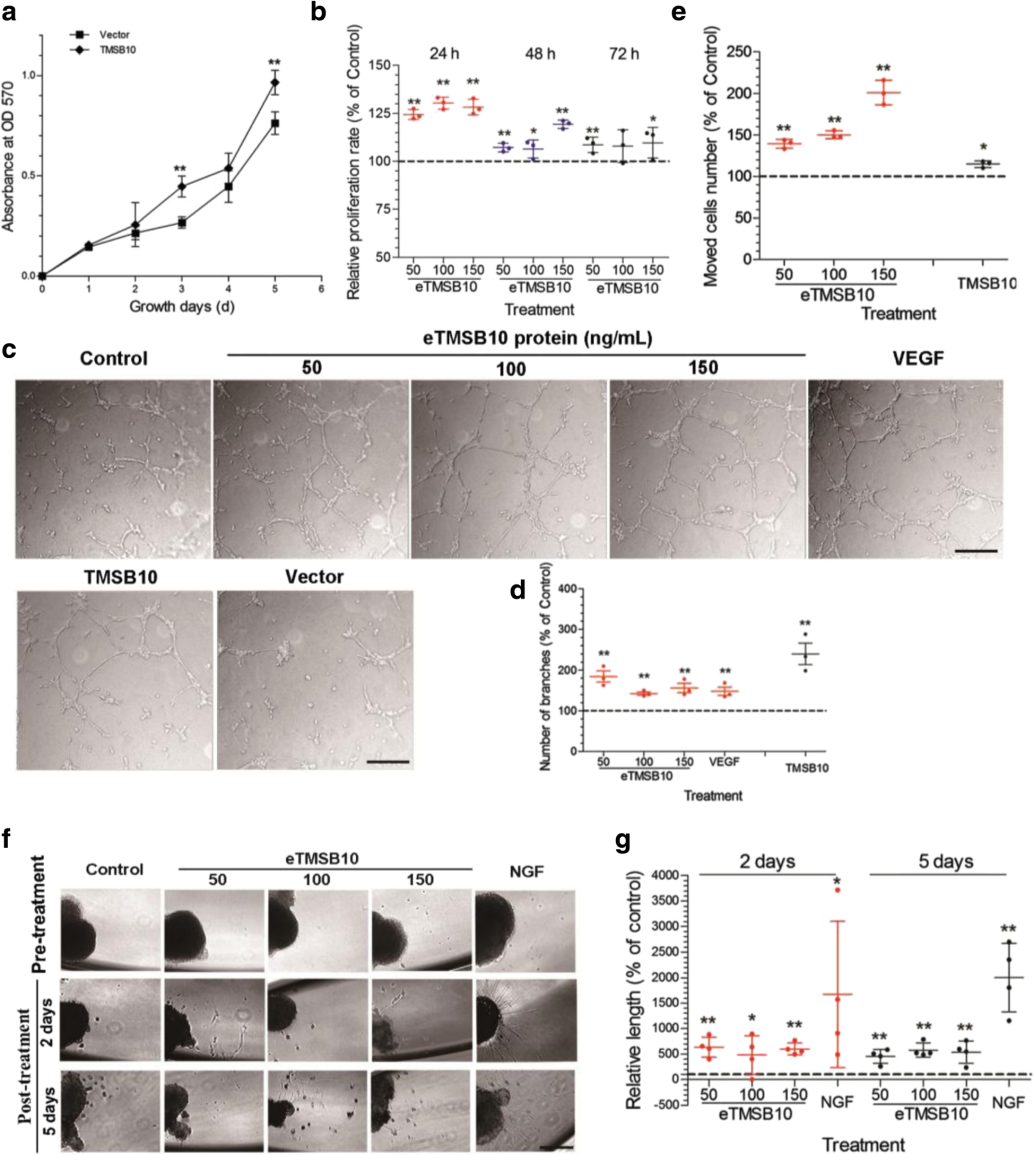
Effects of deer TMSB10 on cell proliferation, tube formation, and motility of HUVECs as well as on DRG outgrowth. **a** HUVECs overexpressing deer TMSB10 compared with vector alone. HUVECs overexpressing deer TMSB10 had significantly higher growth rates than that of the vector control group. **b** Determination of HUVEC proliferation using the MTT assay. HUVEC cells were exposed for 24, 48, and 72 h in the presence of deer exogenous TMSB10 (eTMSB10: 50, 100, or 150 ng/ml) and compared with control. The growth rates of the HUVECs treated with deer eTMSB10 (50, 100, and 150 ng/ml) were significantly higher than in controls. **c** Representative images (inverted phase contrast) of tube formation assays. Scale bars = 200 μm. **d** Quantification of tube formation by calculating the average number of branched vessels per field of view in response to exogenous TMSB10 (eTMSB10: 50, 100, or 150 ng/ml), overexpressed TMSB10 (TMSB10), and vascular endothelial growth factor (VEGF) as a positive control. Results showed that deer eTMSB10 and deer TMSB10 overexpressing HUVECs showed more tube formation in the HUVECs compared with controls or vector. **e** Transwell migration assays with HUVECs treated with deer eTMSB10 (50, 100, and 150 ng/ml) or overexpressing deer TMSB10 were compared with their controls. Results showed that deer eTMSB10 and deer TMSB10 overexpressing HUVECs showed more migration compared with the control or vector. **f** Lamellipodium emerging from a DRG neuron after treatment for 2 days or 5 days with 50, 100, and 150 ng/ml eTMSB10 or nerve growth factor (NGF; 50 ng/ml) as a positive control. Scale bar = 100 μm. **g** Quantification of average length of lamellipodium. Results showed that deer eTMSB10 significantly increased the growth of lamellipodium from DRG compared with the control. Data represent mean ± SD of three or four experiments. *P < 0.05, **P < 0.01, compared with control or vector (control or vector control = 100%). OD optical density

To further investigate the angiogenic potential of deer TMSB10, deer TMSB10 was introduced into HUVECs. Western blot analysis was then performed to measure the protein levels of the deer TMSB10 (Additional file 3: Figure S2; Additional file 9: Figure S8). The growth rates of HUVECs-ODT and HUVECs treated with different concentrations of deer eTMSB10 were examined using an MTT assay. As shown in Fig. 4a, the growth rate of HUVECs-ODT was significantly higher than that of the vector control group (*P* < 0.05). In addition, the growth rates of the HUVECs treated with deer eTMSB10 (50, 100, and 150 ng/ml) were also significantly higher than that of the control (Fig. 4b) (*P* < 0.05 or *P* < 0.01). The in-vitro tube formation study showed more vessels with deer eTMSB10 or deer TMSB10-ODT treatment compared with the controls or the vector (Fig. 4c, d) (*P* < 0.05). The transwell assays showed that HUVECs increased their migration capacity when treated with deer eTMSB10 in a dose-dependent manner from 50 to 150 ng/ml (Fig. 4e) (*P* < 0.05). This result was confirmed using IBIDI cell migration technology in vitro (Additional file 10: Figure S9). Transwell HUVEC migration was also significantly increased in the HUVECs-ODT compared with the vector group (Fig. 4e) (*P* < 0.05). There was no observable difference in cytoskeleton structure between the HUVECs-ODT and the control vector-alone cells (Additional file 11: Figure S10). A DRG outgrowth assay was conducted to investigate the inductive role of deer eTMSB10 in nerve growth in vitro. Growth of lamellipodium from DRG treated with deer eTMSB10 was significantly increased (Fig. 4f, g) (*P* < 0.05). A rat aortic arch assay was conducted to confirm the angiogenic potential of deer eTMSB10 in vitro. The number of buddings from an aortic arch was significantly increased with 50 ng/ml deer eTMSB10 treatment compared with the untreated control group (*P* < 0.05), and a trend was seen for the other concentrations (Additional file 12: Figure S11). Overall, the results indicate that deer TMSB10 promoted tube formation in HUVECs and vascular outgrowth from the aortic arch in vitro, possibly through acceleration of proliferation and migration of cells involved in this process.

### The VEGF family is involved in angiogenesis-promoting roles of deer TMSB10 in HUVECs

**Fig. 5:**
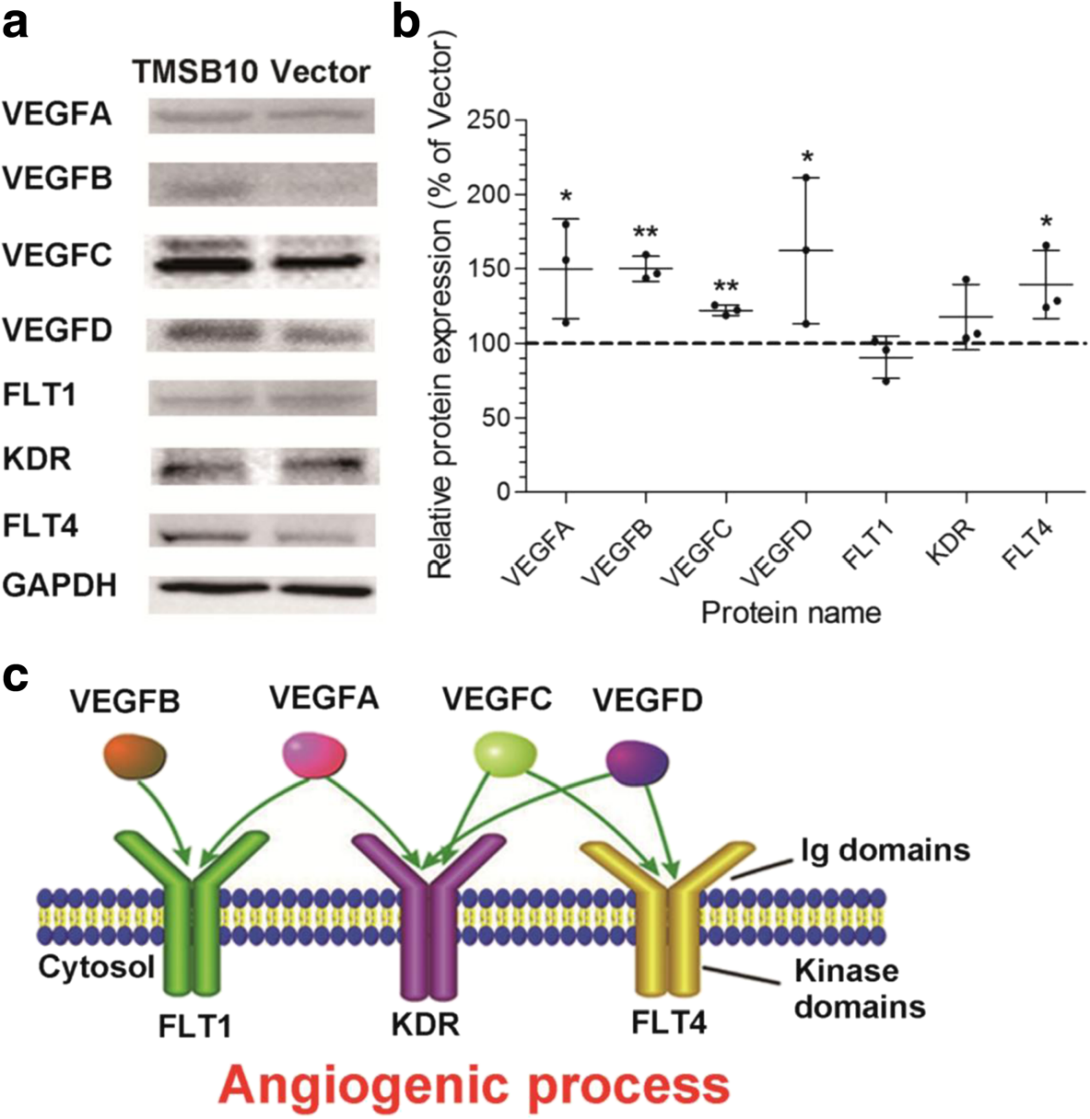
Vascular endothelial growth factor (VEGF) ligands and receptors were found to be essential for the angiogenesis-promoting role of deer TMSB10 in HUVECs. **a** Representative images of VEGFA, VEGFB, VEGFC, VEGFD, FLT1, KDR, and FLT4 protein levels in HUVECs overexpressing deer TMSB10 after Western blot analysis. **b** Quantification of Western blotting for the VEGF family. The protein levels were normalized with GAPDH. The relative levels of proteins were calculated and plotted. The data are expressed as mean ± SD, *n* = 3 experiments. *P < 0.05, **P < 0.01, compared with vector (control set at 100%). **c** VEGF family involved in the angiogenic process

Research was conducted to examine the involvement of angiogenesis-related factors in deer TMSB10 activation of HUVECs. Total protein extracts of HUVECs-ODT were made for immune-blotting to analyze the expression levels of angiogenesis-related proteins compared with the control conditions. Results showed that expression levels of VEGF, VEGF-B, VEGF-C, VEGF-D, and their receptor (VEGFR2 and VEGFR3) were significantly elevated in the HUVECs-ODT compared with the empty vector group (Fig. 5a, b) (*P* < 0.05). It is well known that the VEGF family and their receptors are essential for angiogenesis (Fig. 5c). To further evaluate the signaling pathways of the identified deer TMSB10-related proteins, protein-protein interactions were analyzed based on the experimental evidence, and statistical enrichment tests were carried out using Kyoto Encyclopedia of Genes and Genomes (KEGG) pathway annotations (Additional file 13: Figure S12A). Nine KEGG pathways (*P* < 0.05) were enriched in the dataset using STRING software (Additional file 13: Figure S12B). The most significant pathways for deer TMSB10-related proteins were rap1 pathways, focal adhesion, ras signaling, cytokine-cytokine receptor interaction, and PI3K-akt signaling pathways, which are involved in potentiation of the angiogenic process in HUVECs. Taken together, the VEGF family may be involved in angiogenesis-promoting roles of deer TMSB10 in HUVECs.

### Structural difference between deer and human TMSB10 identified by molecular dynamics simulation

**Fig. 6:**
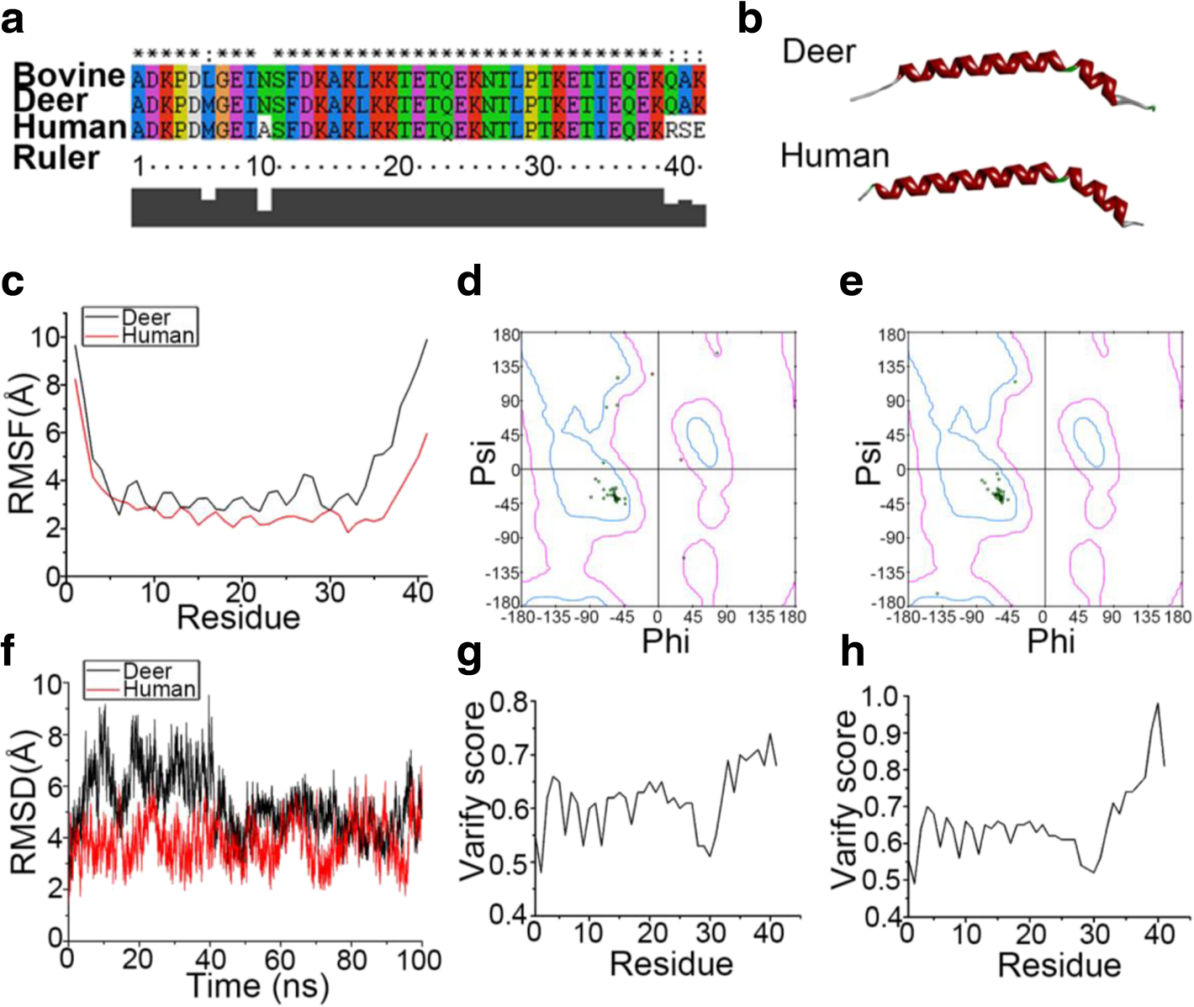
Molecular dynamics simulation and refinement of both deer and human TMSB10 structure. **a** Sequence alignment of TMSB10 (deer and human) and template (bovine). **b** 3D structure of both deer and human TMSB10. In the docked structure, the α-helix (red) and the β-turn (green) are presented. The loop is represented by the color white. **c** The Cα root mean square fluctuation (RMSF) of each residue for both deer (black) and human (red) TMSB10. **d, e** The Ramachandran plot of deer TMSB10 (**d**) and human TMSB10 (**e**) using the Procheck program. The green dots represent residues of TMSB10. The dots surrounded by a curve (cyan) indicate that the residues are in the most favored region. The dots outside the cyan region but in the pink region equate to residues in an additional allowed region (Lys3, Glu37, Gln39, Ala40 for deer TMSB10, and Asp2, Met6 for human TMSB10). The red dots outside the pink region mean the residue is in the disallowed region (Asp2 and Met6 for deer TMSB10). **f** The root mean square deviation (RMSD) of both deer (black) and human (red) TMSB10 during 100 ns MD simulation. **g, h** The evaluation of deer (**g**) and human (**h**) TMSB10 final structure by Profile-3D program. All the scores of residues are above zero; this means the residues are in credible positions

We performed TMSB10 sequence alignment among bovine, deer, and human species. Results showed that both deer and human TMSB10 had good alignment against the template bovine sequence (Fig. 6a). Homology model building was performed using the SWISS-MODEL online. Deer and human TMSB10 both contain two α-helixes, but the angle of the deer protein was smaller than that of human (Fig. 6b). Procheck was used to calculate the percent of backbone θ-φ angles within the allowed Ramachandran region. The final structures were further checked by using Profile-3D and the results are presented in Fig. 6d and e. The Profile-3D method measures the compatibility of amino acid sequence with a known 3D protein structure. When checked using Profile-3D, the self-compatibility scores for deer and human TMSB10 were 25.28 and 26.81, respectively. Note that compatibility scores above zero correspond to an ‘acceptable’ side chain environment. In Fig. 6g and h it is shown that all amino acid residues were logical. The differences between deer TMSB10 and human TMSB10 were further investigated using MD simulation. The Cα root-mean-square fluctuation (RMSF) of deer TMSB10 was higher than that of human TMSB10 (Fig. 6c). Results also showed that the root mean square deviation (RMSD) of the Cα atoms in deer TMSB10 was significantly higher than that in human models before 45 ns (Fig. 6f).

## Discussion

This is the first study to examine the effects of deer TMSB10 (93% amino acid alignment with human TMSB10) on both APCs and HUVECs. In this study, we examined the expression levels of deer TMSB10 in the different layers of the antler growth center and in different antler cell lines. The functions of deer TMSB10 were examined via both the overexpression of deer TMSB10 in the APCs and HUVECs and the treatment of cells with different doses of deer eTMSB10. Deer TMSB10 was found to be a novel promoter for chondrogenesis in vitro and angiogenesis both in vitro and in vivo.

Deer antlers, known to be the only mammalian organs that can achieve annual full regeneration, have valuable potential in the field of regenerative medicine. Antlers possess vascularized cartilage formed through modified endochondral ossification [47, 48]. In this study, expression of deer TMSB10 showed a characteristic distribution (low-high-low) across the antler growth center (from RM, PC, TZ, and to C). TMSB10 was undetectable in the antlerogenic stem cells (APCs and PP cells) and the control FP cells [49]. To test whether deer TMSB10 promotes chondrogenesis and angiogenesis, the APCs (progenitor cells for antler cartilage formation, negative for TMSB10 expression) were either transfected with deer-TMSB10-lentivirus or treated with different doses of deer eTMSB10. Deer TMSB10 was found to promote chondrogenesis in a micromass culture, evidenced by increased cartilage matrix (Alcian Blue staining). The APCs-ODT embedded in Matrigel showed proangiogenic effects in vivo compared with the APCs with empty vector and Matrigel alone. Furthermore, lamellipodium of DRG treated with deer TMSB10 was also significantly increased. Taken together, deer TMSB10 was found to be a novel regulator for antler differentiation, including chondrogenesis, neuronal differentiation, and angiogenesis.

The roles of TMSB10 have been investigated using various human cancer tissues compared with normal tissues in recent years, and it has been identified as a reliable cancer biomarker [50–52]. The molecule has been reported to have various functions, including angiogenesis, but Koutrafouri et al. [46] found that human eTMSB10 inhibited angiogenesis in a CAM assay. To clarify this discrepancy, we performed a CAM assay using both human and deer eTMSB10. Interestingly, we found that deer eTMSB10 significantly increased, while human eTMSB10 decreased, the number of newly formed blood vessels compared with the control. To confirm this result, we also selected another well-established assay, an aortic arch assay, and the results showed that deer eTMSB10 significantly increased tube formation. This is in sharp contrast to the previous report using human TMSB10—i.e., negatively regulating blood vessel formation [46].

Numerous studies show that a small difference in the primary sequence of two closely related proteins (e.g., with a single amino acid substitution) may have distinct effects in biological systems [53–56]. Along this line, we carried out molecular dynamics simulation to understand the reason why deer and human TMSB10 have distinct biological functions. The results showed that human TMSB10 was more stable in molecular structure than deer TMSB10. The higher flexibility of deer TMSB10 in its molecular structure may provide better opportunities for it to interact with other functional proteins. In addition, bovine TMSB10 (also named TMSB9), with a single amino acid substitution when compared with deer TMSB10, was found to inhibit angiogenesis in vivo on a CAM model [46]. This is understandable since to alter the functions of TMSB10 from inhibition (human) to stimulation (deer) in angiogenesis and chondrogenesis would greatly facilitate the growth of deer antlers, arguably the fastest growing mammalian tissue in nature (it can reach growth rates up to 2 cm/day [57]).

It is reported that the effects of TMSB10 on angiogenesis may be mediated via VEGF pathways. Overexpression of human TMSB10 is found to markedly inhibited VEGF-induced endothelial tube formation in vitro, vessel sprouting ex vivo [58], and tube formation in hypoxia-induced monkey choroid-retinal endothelial (RF/6A) cells [59]. In addition, human eTMSB10 was found to inhibit human coronary artery endothelial cell (HCAEC) migration and tube formation [15]. In the present study, Western blotting analysis was used to separate and identify the potential molecules that may mediate the induction of angiogenesis of deer TMSB10. Levels of the angiogenesis-related proteins VEGF, VEGF-B, VEGF-C, VEGF-D, and their receptors VEGFR2 and VEGFR3 were all elevated in HUVECs overexpressing deer TMSB10. These results are in accord with the previous reports that human recombinant TMSB10 promoted VEGF-C expression in lung cancer cells [60], and that recombinant VEGF treatment elevated the expression level of human TMSB10 in endothelial cells [61]. Others have, however, reported that overexpression of human TMSB10 inhibited VEGF mRNA expression and autocrine VEGF protein production in RF/6A cells [59]. Taken together, these results indicate that deer TMSB10 is involved in the upregulation of proteins of the VEGF family, as well as the VEGF receptors (VEGFR2 and VEGFR3) in angiogenesis using HUVECs.

TMSB10 has strong angiogenic and chondrogenic properties and is a small molecule (42 amino acids) that belongs to the category of polypeptides (< 51 amino acids) [62]. Polypeptides generally have low molecular weight (thus are relatively stable), low immunogenicity, low cytotoxicity, and high activity [63]. Therefore, TMSB10 should have great developmental potential as a therapeutic in clinics for enhancing the healing of chronic wounds that are mainly caused by the failure of blood vessels to grow (such as diabetic foot) or cartilage repair (such as osteoarthritis). Indeed, recently we applied TMSB10 to cartilage repair using rabbit articular cartilage defects as a model. The preliminary results are very encouraging; the artificially created cartilage defects were fully filled with typical cartilage tissue in the group with the addition of TMSB10, whereas they were only partially filled with the mixture of fibrous and cartilaginous tissues in the group without the presence of TMSB10 (unpublished data). We believe that, with further optimization of the application procedure and dosage, treatment of articular cartilage defects using TMSB10 can be extended from laboratory animals to clinical trials.

**Fig. 7:**
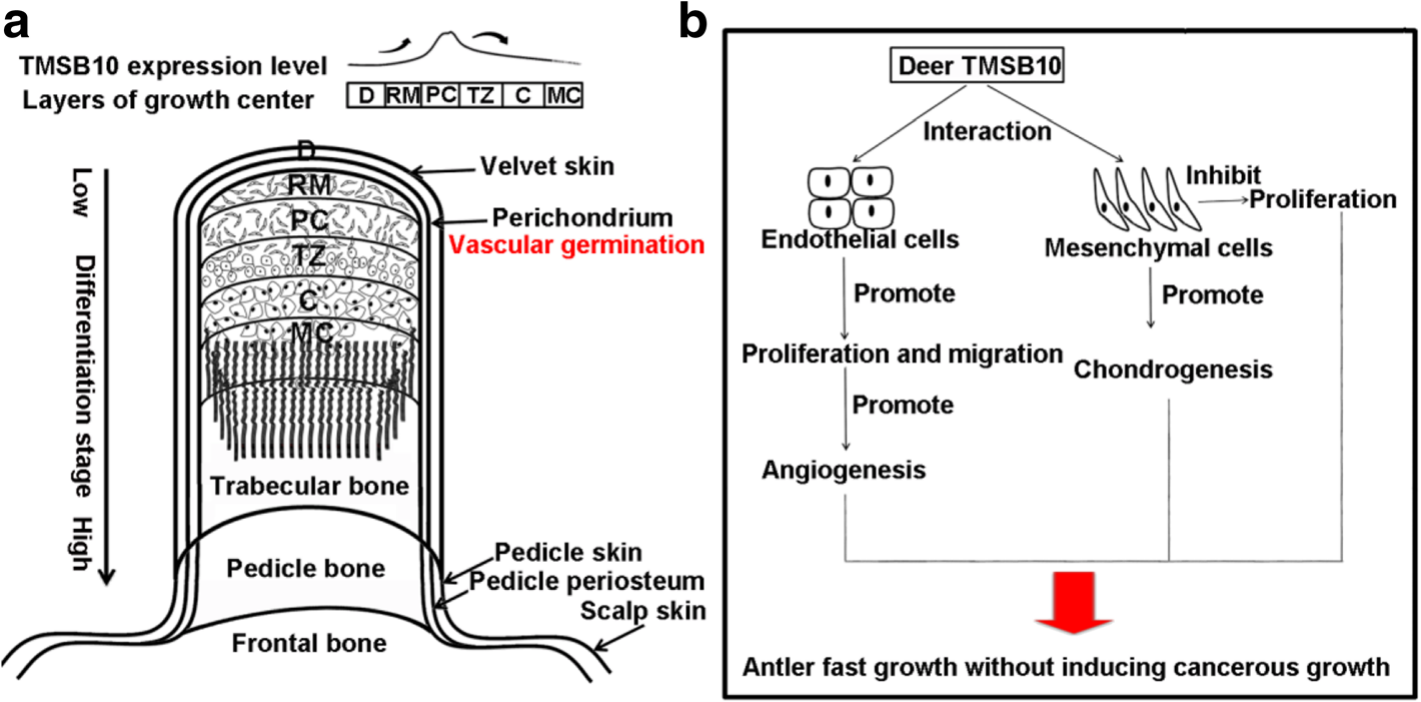
Schematic diagram depicting the involvement of deer TMSB10 during antler growth. **a** TMSB10 expression levels in the different layers of the growing antler, the distribution of blood vessels, and the structure of an antler. **b** Diagram of potential mechanisms of TMSB10 involvement in rapid antler growth without inducing cancer. Deer TMSB10 interacted with endothelial cells to promote proliferation and migration, ultimately leading to angiogenesis. TMSB10 promoted chondrogenesis and suppressed the proliferation of AP cells. C cartilage, D dermis, MC mineralized cartilage, PC precartilage, RM reserve mesenchyme, TZ transitional zone

In summary, the location, expression level, and expression pattern of deer TMSB10 in the antler growth center indicates that TMSB10 may be involved in chondrogenesis and angiogenesis. Functional analysis conducted here supports the hypothesis that deer TMSB10 enhances cartilage differentiation, neuronal differentiation, and angiogenesis. This is the first report that deer TMSB10 functions as a novel stimulating factor for cartilage differentiation and angiogenesis in the process of antler growth, a novel model for investigating stem cell-based organ regeneration (Fig. 7).

## Conclusion

Deer TMSB10, a 42-amino acid protein, is widely expressed in the antler growth center, with highest expression in the PC layer where angiogenesis occurs. In this study, both exogenous deer TMSB10 (eTMSB10) and transfected deer TMSB10 had negative impacts on the proliferation of APCs, while positively inducing the proliferation of HUVECs. Deer TMSB10 promoted chondrogenesis in a micromass culture using the APCs, and angiogenesis in CAM, HUVEC tube formation, and aortic ring assays. In conclusion, deer TMSB10 was found to be a novel stimulus of angiogenesis and chondrogenesis during antler growth and/or regeneration.

## Additional files

Additional file 1: **Figure S1.** Tissue location of primary cultured cells. (Reproduced from our previous works [22] with permission from the *Journal of Agricultural Biotechnology*). AP: antlerogenic periosteum; tissue from which deer pedicle and initial antler develops. PP: pedicle periosteum; tissue which directly forms from the AP and gives rise to subsequent regenerating antlers. FP: facial periosteum; periosteum with no capacity to generate antler; D: dermis; dermal connective tissue and epidermis of growing antler. RM: reserve mesenchyme; growth center of antler containing the stem cells. PC: precartilage; aligned blood vessels separated by precartilage cells in the growing antler. TZ: transition zone; transitional area. C: cartilage; vascularized cartilage in growing antler. Antler stem cells (ASCs) have been found to reside within the AP, PP, and RM zones. Chondrogenesis occurs progressively in the RM, PC, TZ, and C, and these layers are central for studying cartilage formation. (PDF 127 kb)

Additional file 2: **Table S1.** Primers. (DOC 37 kb)

Additional file 3: **Figure S2.** Infection of antlerogenic periosteum and human umbilical vein endothelial cells using recombination lentivirus. (A) Antlerogenic periosteum (AP) cells and (B) human umbilical vein endothelial cells (HUVEC) were trypsinized and resuspended in the lentiviral vector supernatants (TMSB10 and vector) to a concentration at 2.5 × 10^4^ cells/ml (5 × 10^4^ for HUVECs) in six-well plates. Cells were enriched by puromycin selection for 2 weeks and visualized by fluorescence microscope. RFP, red fluorescence protein-TMSB10; PH, phase contrast. Scale bar = 200 μm. (Figure S2A is reprinted from our previous works [25] Copyright (2017), with permission from the *Journal of Agricultural Biotechnology*). (PDF 514 kb)

Additional file 4: **Figure S3.** Concentration of deer and human exogenous TMSB10 (eTMSB10) as determined by HPLC postpeptide synthesis. (A) Deer eTMSB10 at 98.2469%. (B) Human eTMSB10 at 98.1311. (PDF 294 kb)

Additional file 5: **Figure S4.** Detection and characterization of deer TMSB10. (A) PCR amplification of deer TMSB10 (129 bp) amplified from the total mRNA of reserve mesenchyme cells. (B) Predicted three-dimensional (3D) structural model of deer TMSB10. The 3D model was obtained using the SWISS-MODEL server. (C) Alignment of TMSB10 amino acid sequence with human, mouse, bovine, and sheep (Gene bank accession no. NP_066926.1, NP_001034481.1, NP_777048.1, XP_017910878.1, respectively). The cervine (deer) TMSB10 sequence aligns has 93%, 93%, 99%, and 100% identity to the human, mouse, bovine, and sheep, respectively. Asterisks show the same amino acid residue; Arrows show potential phosphorylation sites. (PDF 84 kb)

Additional file 6: **Figure S5.** Expression level of deer TMSB10 in antlerogenic periosteum (AP) cells induced using a lentivirus overexpression system. Expression level of deer TMSB10 protein using Western blotting assay. TMSB10: AP cells with the deer TMSB10 vector; Vector: AP cells carried empty vector. GAPDH as control. (Reproduced from our previous works [25] Copyright (2017), with permission from the *Journal of Agricultural Biotechnology*). (PDF 94 kb)

Additional file 7: **Figure S6.** Effects of deer eTMSB10 on migration of antlerogenic periosteum cells in vitro. (A) Representative images of the migration assay. (B) Quantitation of the cells which migrated into the boxed space with each condition. The time taken by the cells to fill the gap was observed under an inverted microscope and measurements take after 24 h. Results are reported as the mean ± SD, *n* = 3 independent experiments. Control = 100%. *P < 0.05, **P < 0.01. (PDF 1334 kb)

Additional file 8: **Figure S7.** Concentration of TNFα in the culture medium of antlerogenic periosteum cells of deer overexpressing TMSB10 or with empty vector. This study was performed using an ELISA according to the manufacturer’s instructions. 1.0 × 10^5^ cells were seeded in six-well plates and cultured at 37 °C under 5% CO_2_ for 24 h. The culture medium was collected and stored at −20 °C for analysis. *P < 0.05. (PDF 19 kb)

Additional file 9: **Figure S8.** Expression of deer TMSB10 by human umbilical vein endothelial cells (HUVECs) induced by lentivirus. Expression level of deer TMSB10 protein using Western blotting assay. TMSB10: HUVECs with deer TMSB10 vector, Vector: HUVECs with empty vector. GAPDH as control. (PDF 15 kb)

Additional file 10: **Figure S9.** Effects of eTMSB10 on the migration of human umbilical vein endothelial cells in vitro. The number cells to migrate into the gap (boxed area) was observed under an inverted microscope after 24 h of exposure to treatment or control conditions. (A) Representative images. (B) Quantitation of the number of cells within the boxed area. Results are reported as the mean ± SD, *n* = 3 independent experiments. Control = 100%. *P < 0.05, **P < 0.01. (PDF 1404 kb)

Additional file 11: **Figure S10.** Human umbilical vein endothelial cells overexpressing deer TMSB10 or with vector alone are labeled with microtubulin (green). Scale bar = 100 μm. (PDF 91 kb)

Additional file 12: **Figure S11.** Effects of deer exogenous TMSB10 (eTMSB10) on the formation of capillary-like budding in the mouse aortic arch with VEGF as a positive control. (A) Representative images of budding by the aortic arches. (B) Quantification of number of buddings. Data represent mean ± SD, *n* =3 experiments. Control = 100%. Scale bar = 250 μm. *P < 0.05, **P < 0.01. (PDF 70 kb)

Additional file 13: **Figure S12.** Interaction of deer TMSB10-related proteins and distribution of KEGG (Kyoto Encyclopedia of Genes and Genomes) pathways in which the deer TMSB10-related proteins participate. (A) Protein interactions. (B) Enrichment of pathways. KEGG pathways are arranged in ascending order according to the *P* values. Dash line represents *P* < 0.01 or *P* < 0.05. (PDF 72 kb)

## Abbreviations

AP: Antlerogenic periosteum
APC: Antlerogenic periosteal cell
bFGF: Basic fibroblast growth factor
BSA: Bovine serum albumin
C: Cartilage
CAM: Chicken embryo chorioallantoic membrane
D: Dermis
DMEM: Dulbecco’s modified Eagle’s medium
DMSO: Dimethyl sulfoxide
DRG: Dorsal root ganglia
FBS: Fetal bovine serum
FP: Facial periosteum
HGF: Hepatocyte growth factor
HRP: Horseradish peroxidase
HUVEC: Human umbilical vein endothelial cell
MD: Molecular dynamics
NGF: Nerve growth factor
OD: Optical density
ODT: Overexpressing deer TMSB10
PBS: Phosphate-buffered saline
PC: Precartilage
PP: Pedicle periosteum
RM: Reserve mesenchyme
RT-PCR: Reverse transcription polymerase chain reaction
SD: Standard deviation
TGF: Transforming growth factor
TMSB: Thymosin beta
TZ: Transitional zone
VEGF: Vascular endothelial growth factor
