# Supplementary figures and images for: Deer thymosin beta 10 functions as a novel factor for angiogenesis and chondrogenesis during antler growth and regeneration
Source: Stem Cell Res Ther. 2018 Jun 19;9:166. doi: 10.1186/s13287-018-0917-y (PMC6009950; doi:10.1186/s13287-018-0917-y)

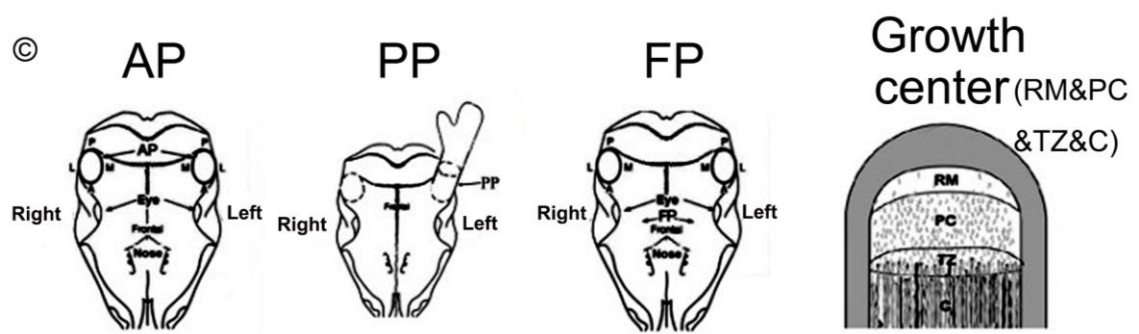

Additional file 1: Figure S1

Supplement: Supplementary file 1 — Figure S1. Tissue location of primary cultured cells. (Reproduced from our previous works [22] with permission from the Journal of Agricultural Biotechnology). AP: antlerogenic periosteum; tissue from which deer pedicle and initial antler develops. PP: pedicle periosteum; tissue which directly forms from the AP and gives rise to subsequent regenerating antlers. FP: facial periosteum; periosteum with no capacity to generate antler; D: dermis; dermal connective tissue and epidermis of growing antler. RM: reserve mesenchyme; growth center of antler containing the stem cells. PC: precartilage; aligned blood vessels separated by precartilage cells in the growing antler. TZ: transition zone; transitional area. C: cartilage; vascularized cartilage in growing antler. Antler stem cells (ASCs) have been found to reside within the AP, PP, and RM zones. Chondrogenesis occurs progressively in the RM, PC, TZ, and C, and these layers are central for studying cartilage formation. (PDF 127 kb) [file 13287_2018_917_MOESM1_ESM.pdf]

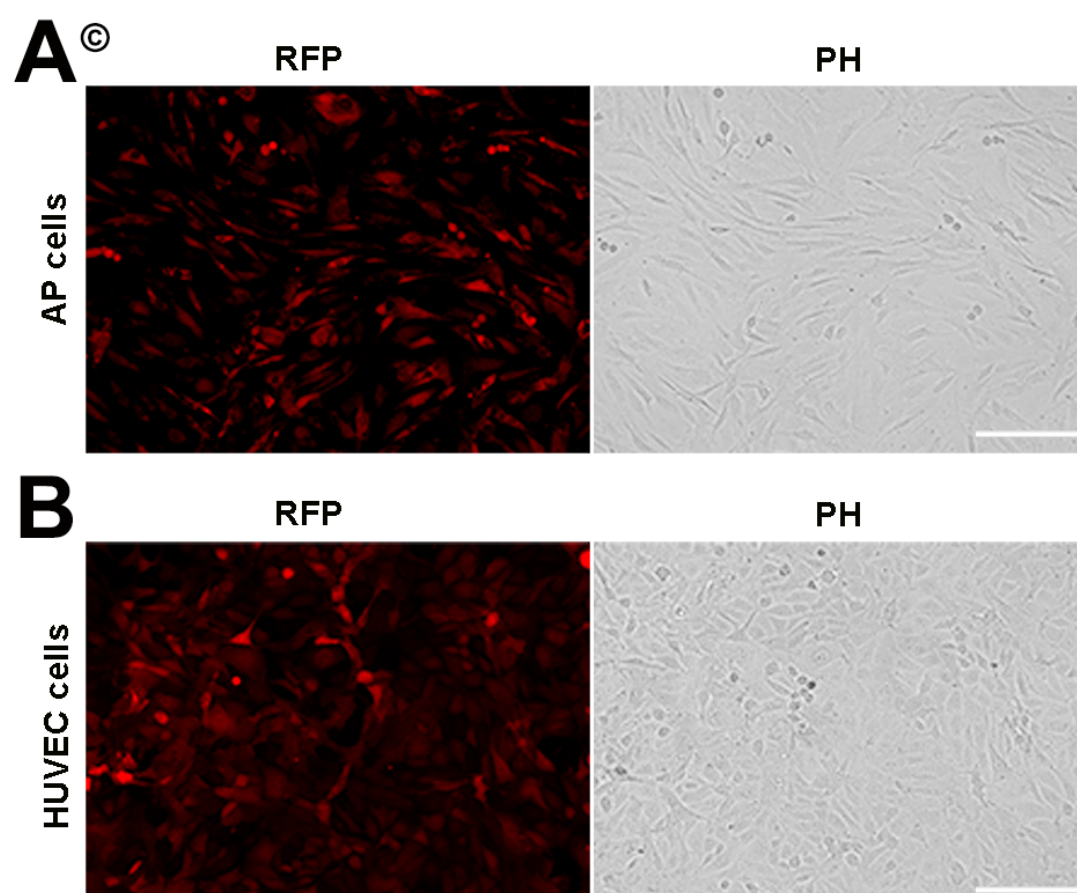

Additional file 3: Figure S2

Supplement: Supplementary file 3 — Figure S2. Infection of antlerogenic periosteum and human umbilical vein endothelial cells using recombination lentivirus. (A) Antlerogenic periosteum (AP) cells and (B) human umbilical vein endothelial cells (HUVEC) were trypsinized and resuspended in the lentiviral vector supernatants (TMSB10 and vector) to a concentration at 2.5 × 10^4^ cells/ml (5 × 10^4^ for HUVECs) in six-well plates. Cells were enriched by puromycin selection for 2 weeks and visualized by fluorescence microscope. RFP, red fluorescence protein-TMSB10; PH, phase contrast. Scale bar = 200 μm. (Figure S2A is reprinted from our previous works [25] Copyright (2017), with permission from the Journal of Agricultural Biotechnology). (PDF 514 kb) [file 13287_2018_917_MOESM3_ESM.pdf]

**A**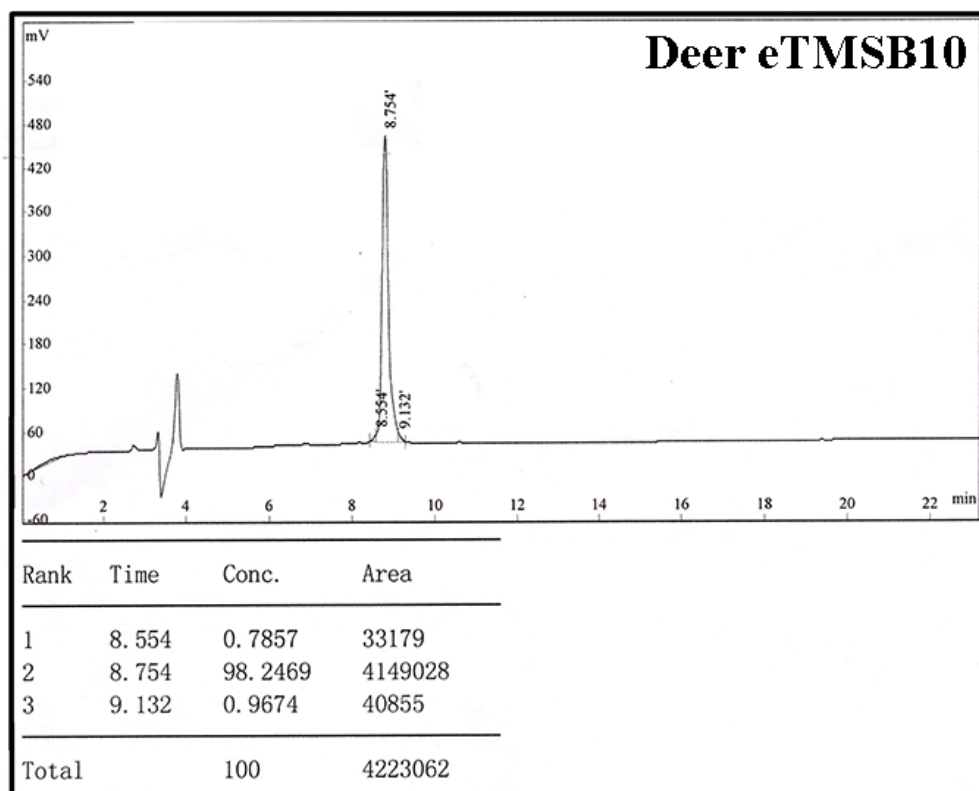**B**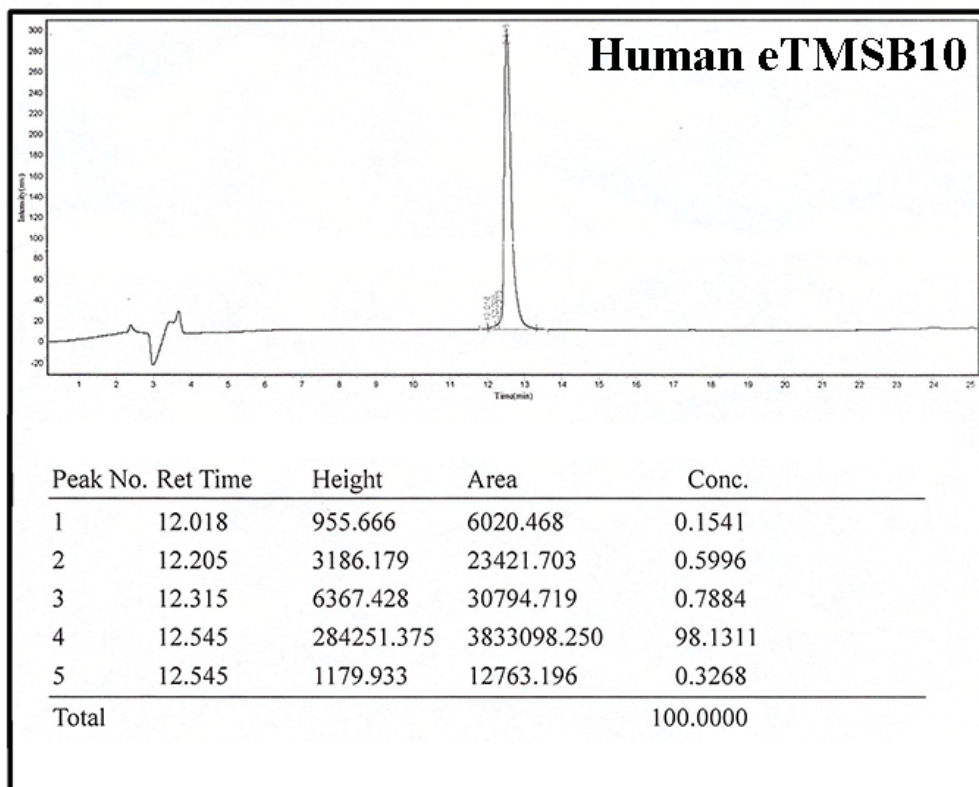

Additional file 4:Figure S3

Supplement: Supplementary file 4 — Figure S3. Concentration of deer and human exogenous TMSB10 (eTMSB10) as determined by HPLC postpeptide synthesis. (A) Deer eTMSB10 at 98.2469%. (B) Human eTMSB10 at 98.1311. (PDF 294 kb) [file 13287_2018_917_MOESM4_ESM.pdf]

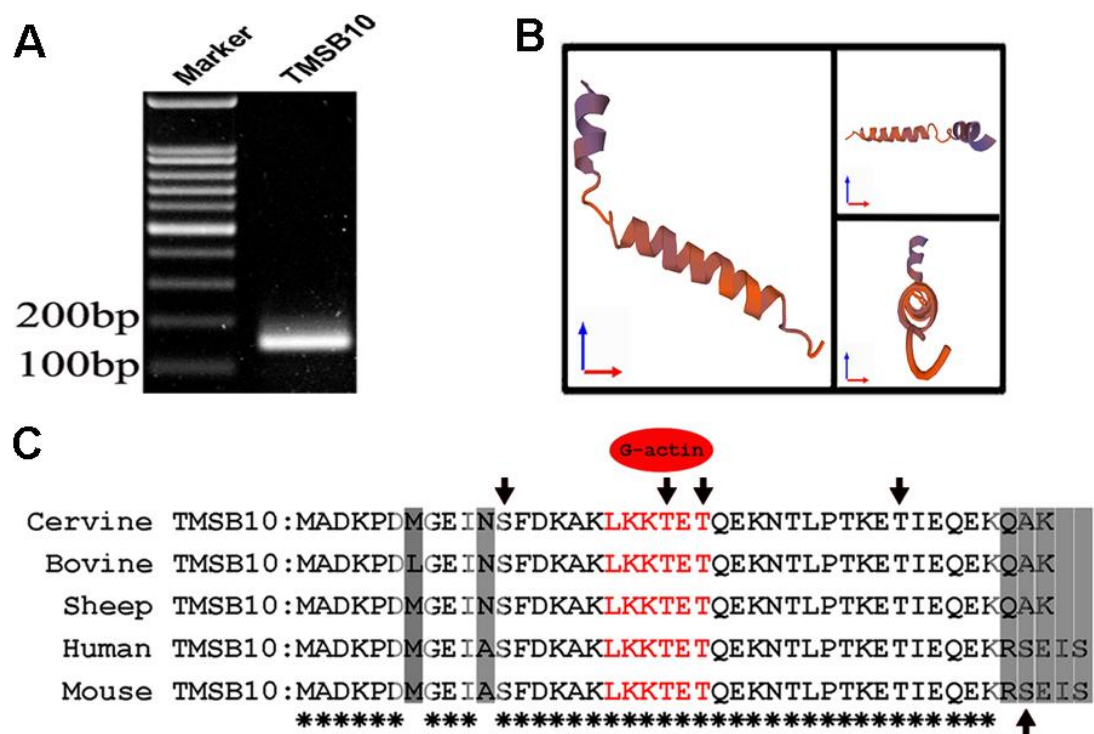

Additional file 5:Figure S4

Supplement: Supplementary file 5 — Figure S4. Detection and characterization of deer TMSB10. (A) PCR amplification of deer TMSB10 (129 bp) amplified from the total mRNA of reserve mesenchyme cells. (B) Predicted three-dimensional (3D) structural model of deer TMSB10. The 3D model was obtained using the SWISS-MODEL server. (C) Alignment of TMSB10 amino acid sequence with human, mouse, bovine, and sheep (Gene bank accession no. NP_066926.1, NP_001034481.1, NP_777048.1, XP_017910878.1, respectively). The cervine (deer) TMSB10 sequence aligns has 93%, 93%, 99%, and 100% identity to the human, mouse, bovine, and sheep, respectively. Asterisks show the same amino acid residue; Arrows show potential phosphorylation sites. (PDF 84 kb) [file 13287_2018_917_MOESM5_ESM.pdf]

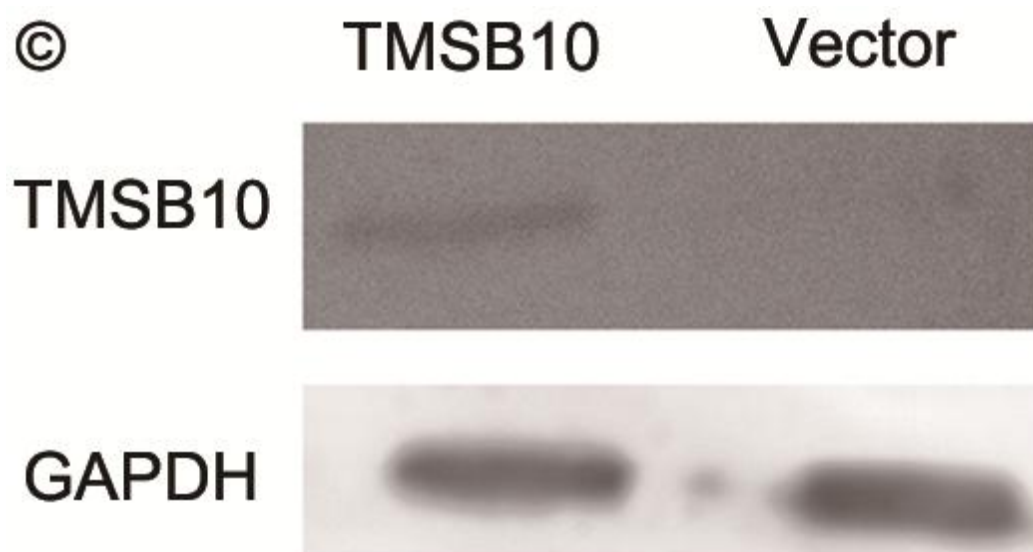

Additional file 6: Figure S5

Supplement: Supplementary file 6 — Figure S5. Expression level of deer TMSB10 in antlerogenic periosteum (AP) cells induced using a lentivirus overexpression system. Expression level of deer TMSB10 protein using Western blotting assay. TMSB10: AP cells with the deer TMSB10 vector; Vector: AP cells carried empty vector. GAPDH as control. (Reproduced from our previous works [25] Copyright (2017), with permission from the Journal of Agricultural Biotechnology). (PDF 94 kb) [file 13287_2018_917_MOESM6_ESM.pdf]

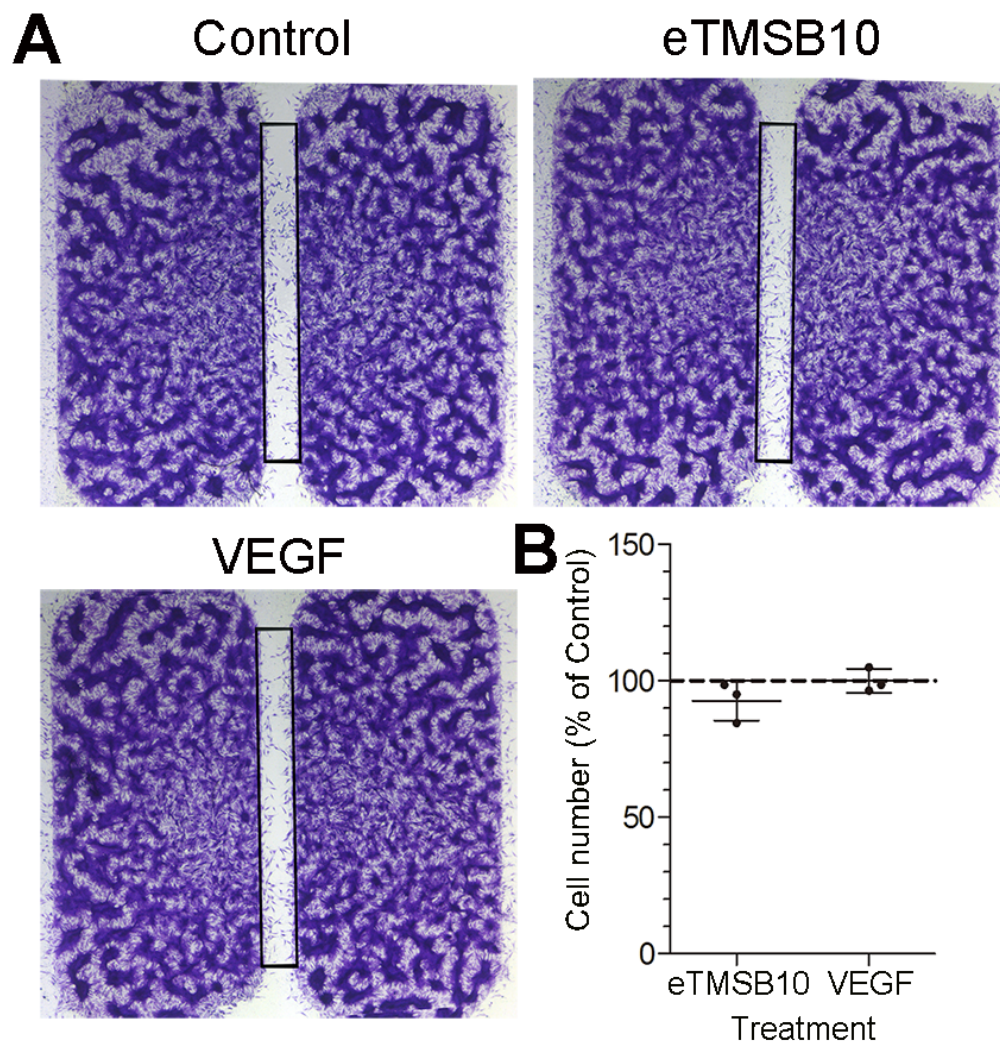

Additional file 7: Figure S6

Supplement: Supplementary file 7 — Figure S6. Effects of deer eTMSB10 on migration of antlerogenic periosteum cells in vitro. (A) Representative images of the migration assay. (B) Quantitation of the cells which migrated into the boxed space with each condition. The time taken by the cells to fill the gap was observed under an inverted microscope and measurements take after 24 h. Results are reported as the mean ± SD, n = 3 independent experiments. Control = 100%. *P < 0.05, **P < 0.01. (PDF 1334 kb) [file 13287_2018_917_MOESM7_ESM.pdf]

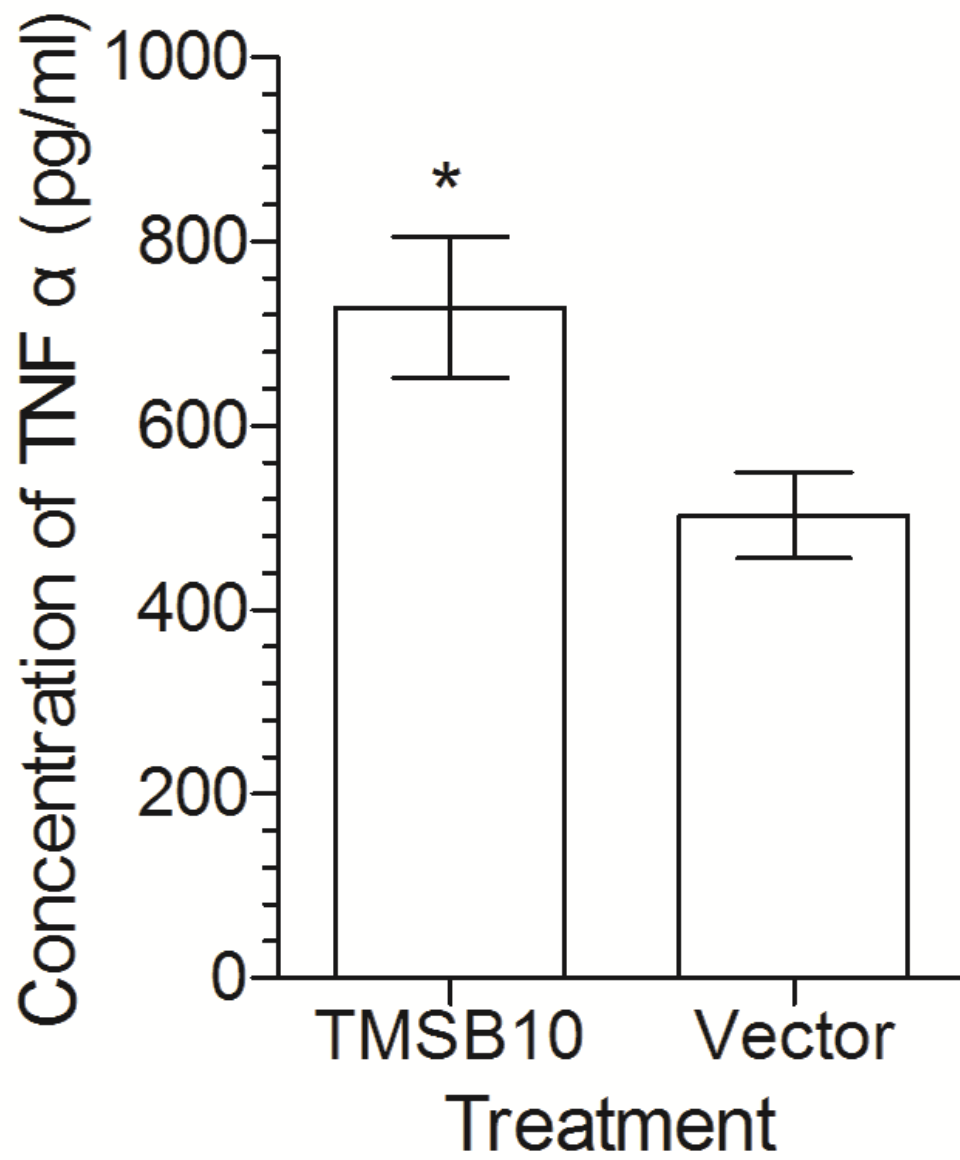

Additional file 8:Figure S7

Supplement: Supplementary file 8 — Figure S7. Concentration of TNFα in the culture medium of antlerogenic periosteum cells of deer overexpressing TMSB10 or with empty vector. This study was performed using an ELISA according to the manufacturer’s instructions. 1.0 × 10^5^ cells were seeded in six-well plates and cultured at 37 °C under 5% CO2 for 24 h. The culture medium was collected and stored at −20 °C for analysis. *P < 0.05. (PDF 19 kb) [file 13287_2018_917_MOESM8_ESM.pdf]

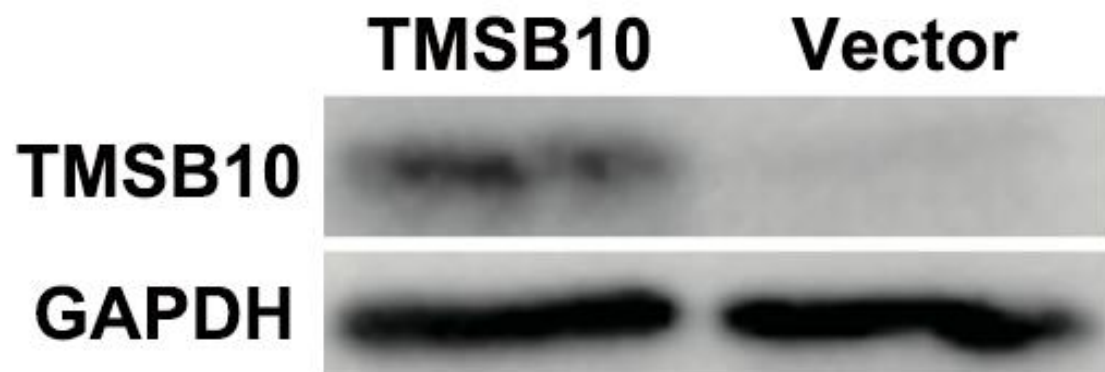

Additional file 9:Figure S8

Supplement: Supplementary file 9 — Figure S8. Expression of deer TMSB10 by human umbilical vein endothelial cells (HUVECs) induced by lentivirus. Expression level of deer TMSB10 protein using Western blotting assay. TMSB10: HUVECs with deer TMSB10 vector, Vector: HUVECs with empty vector. GAPDH as control. (PDF 15 kb) [file 13287_2018_917_MOESM9_ESM.pdf]

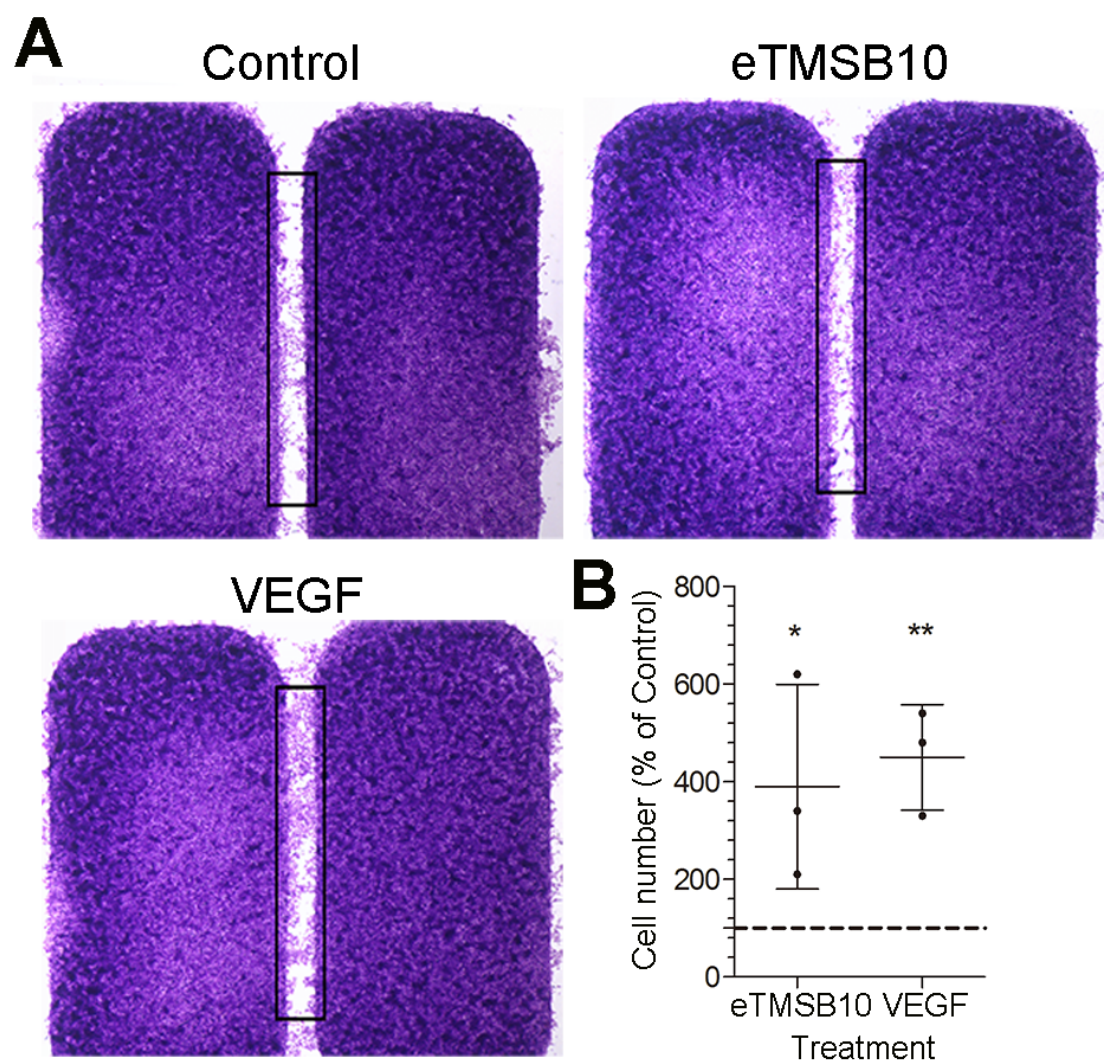

Additional file 10:Figure S9

Supplement: Supplementary file 10 — Figure S9. Effects of eTMSB10 on the migration of human umbilical vein endothelial cells in vitro. The number cells to migrate into the gap (boxed area) was observed under an inverted microscope after 24 h of exposure to treatment or control conditions. (A) Representative images. (B) Quantitation of the number of cells within the boxed area. Results are reported as the mean ± SD, n = 3 independent experiments. Control = 100%. *P < 0.05, **P < 0.01. (PDF 1404 kb) [file 13287_2018_917_MOESM10_ESM.pdf]

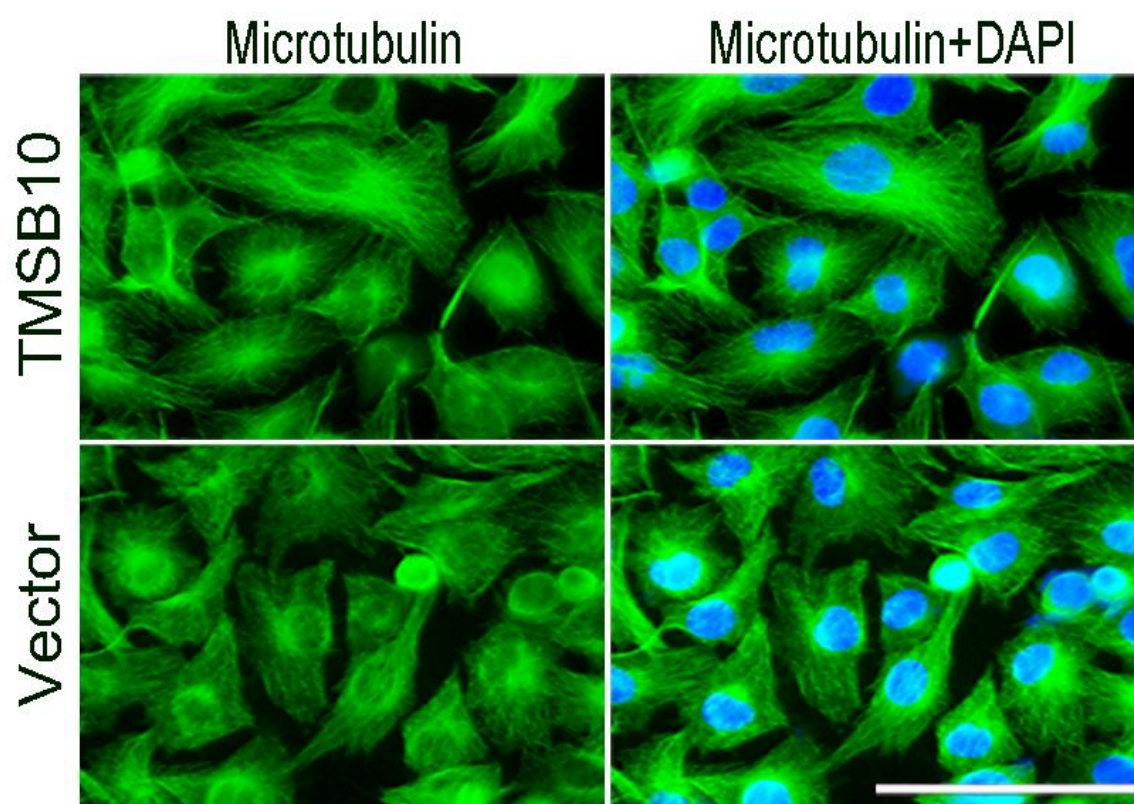

Additional file 11:Figure S10

Supplement: Supplementary file 11 — Figure S10. Human umbilical vein endothelial cells overexpressing deer TMSB10 or with vector alone are labeled with microtubulin (green). Scale bar = 100 μm. (PDF 91 kb) [file 13287_2018_917_MOESM11_ESM.pdf]

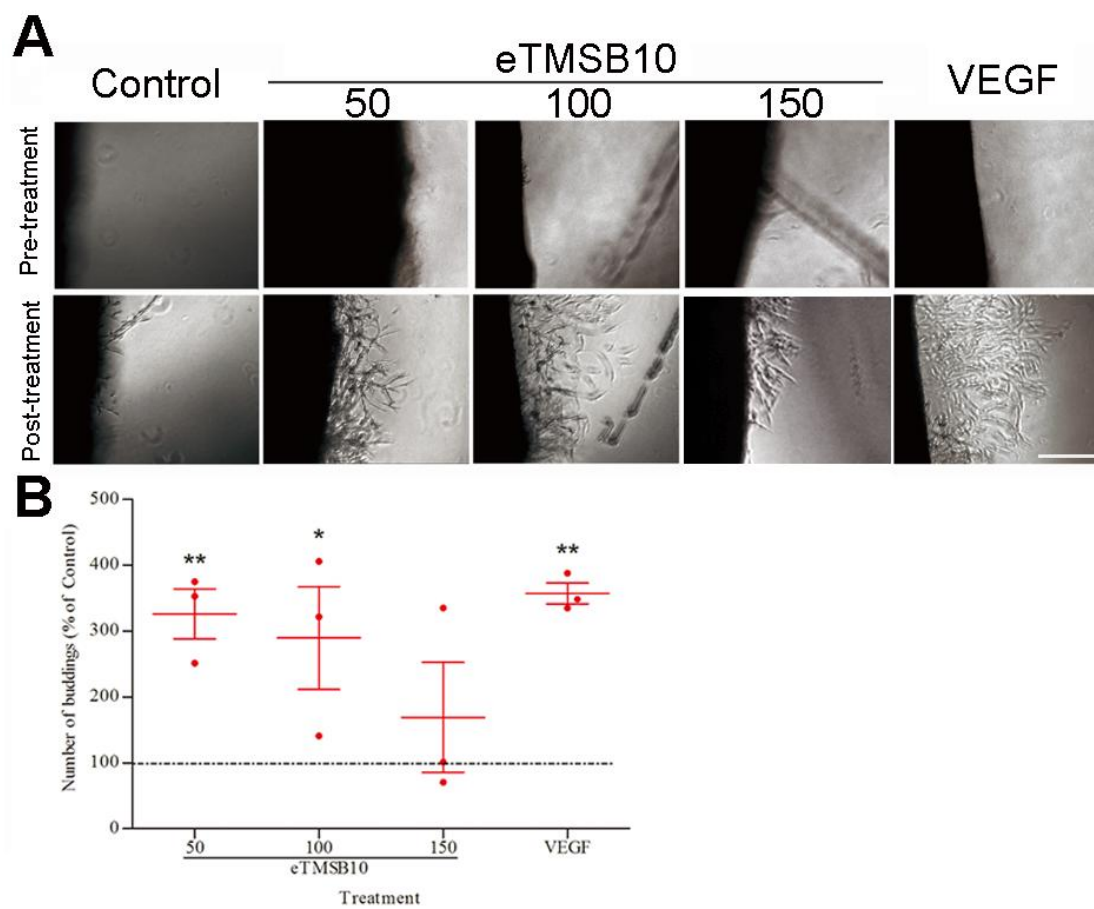

Additional file 12:Figure S11

Supplement: Supplementary file 12 — Figure S11. Effects of deer exogenous TMSB10 (eTMSB10) on the formation of capillary-like budding in the mouse aortic arch with VEGF as a positive control. (A) Representative images of budding by the aortic arches. (B) Quantification of number of buddings. Data represent mean ± SD, n =3 experiments. Control = 100%. Scale bar = 250 μm. *P < 0.05, **P < 0.01. (PDF 70 kb) [file 13287_2018_917_MOESM12_ESM.pdf]

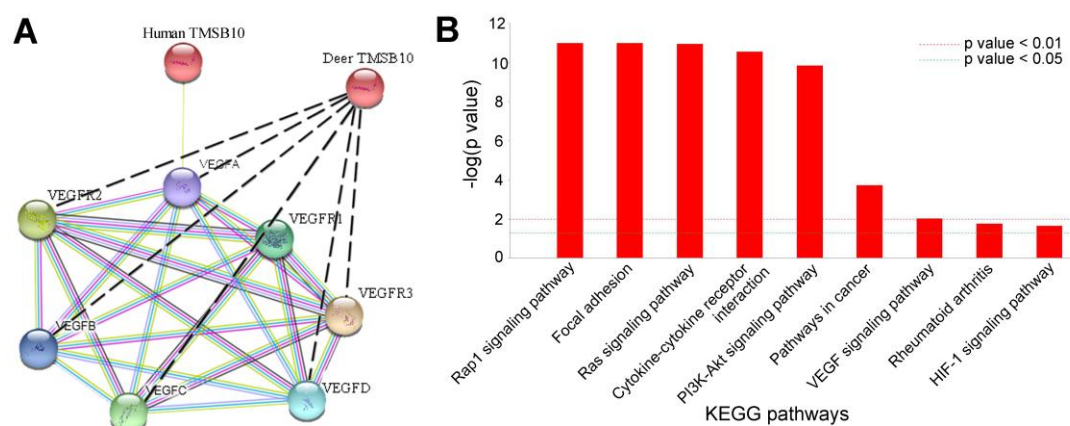

Additional file 13:Figure S12

Supplement: Supplementary file 13 — Figure S12. Interaction of deer TMSB10-related proteins and distribution of KEGG (Kyoto Encyclopedia of Genes and Genomes) pathways in which the deer TMSB10-related proteins participate. (A) Protein interactions. (B) Enrichment of pathways. KEGG pathways are arranged in ascending order according to the P values. Dash line represents P < 0.01 or P < 0.05. (PDF 72 kb) [file 13287_2018_917_MOESM13_ESM.pdf]
