# Supplementary material for: Deer thymosin beta 10 functions as a novel factor for angiogenesis and chondrogenesis during antler growth and regeneration
Source: Stem Cell Res Ther. 2018 Jun 19;9:166. doi: 10.1186/s13287-018-0917-y (PMC6009950; doi:10.1186/s13287-018-0917-y)
Supplement: Supplementary file 2 — Table S1. Primers. (DOC 37 kb) [file 13287_2018_917_MOESM2_ESM.doc]

**Table S1 Primers**

| **Gene name** | **Forward** | **Reverse** | **Gene bank No.** | **Product length** | **Tm/℃** |
| --- | --- | --- | --- | --- | --- |
| **Deer cells** |  |  |  |  |  |
| TMSB10-RT | ATGGCAGACAAGCCCGACATGG | TCACTTTGCTTGCTTCTCCTGCTC | XM_020889419.1 | 129 | 55.5 |
| TMSB10-Clone | TACCGGACTCAGATCTCGAATGGCAGACAAGCCCGACAT | AATACCGGAGTACTCGATCACTTTGCTTGCTTCTCCTGCTC | XM_020889419.1 | 162 | 56.5 |
|  |  |  |  |  |  |
| **Vector** |  |  |  |  |  |
| Plvx-puro | GTCGTAACAACTCCGCC | CGGGGAAACAGAAGTGC | / | 315 | 54.6 |

TMSB10-Clone: recombination site of the vector has been shaded.
